# Real-Time stability enhancement of DDPMSG-based tidal hybrid power systems using heuristic optimization

**DOI:** 10.1038/s41598-026-42638-1

**Published:** 2026-03-08

**Authors:** Javed Khan Bhutto, Asit Mohanty, Pragyan P. Mohanty, Abhaya S. Satpathy, Soumya Ranjan Das

**Affiliations:** 1 Department of Electrical Engineering, King Khalid University, Abha, Saudi Arabia; 2 Institute of Power Engineering, National Energy University, Kualalumpur, 43000 Malaysia; 3 Centre for Promotion of Research, Graphic Era (Deemed to be University), Clementtown, Dehradun India; 4 Department of Mechanical Engineering, VSSUT, Burla, India; 5 Faculty of management studies, Sri Sri University, Cuttack, India; 6 Manipal Institute of Technology, Manipal Academy of Higher Education, Manipal, India

**Keywords:** DDPMSG, DEA, FA, Hybrid FA, Stability, Reactive power compensation, UPFC, Energy science and technology, Engineering, Mathematics and computing

## Abstract

In this research, we analyze the optimal configuration and stability issues associated with a tidal hybrid power system employing a Direct Drive Permanent Magnet Synchronous Generator (DDPMSG). The Tidal system has become unstable since the natural tidal pattern and wind power input have been disturbed. The use of a controller for the Unified Power Flow Controller (UPFC) makes the system stable. Stability analysis, which uses Eigen and Nyquist plots, is used to see how well the proposed controllers work. Additionally, it is evident that the regulator can be effectively calibrated when the variables significantly influence the system’s performance. So, to find the best output for the suggested controller, it is best to use heuristic optimization methods like the Differential Evolution Algorithm (DEA) and the Firefly Algorithm (FA), followed by hybrid FA. The results indicate that the hybrid FA-based system demonstrates enhanced stability, evidenced by increased performance in settling time, rising time, peak overshoot, and damping. The performance and durability assessment of the controller in question is executed using real-time data via OPAL-RT 5142, a digital simulation platform tailored for real-time applications.

## Introduction

The deregulation of the energy market has sped up the use of new, renewable, and decentralized power production technologies, which are often called distributed generation (DG).RESs naturally produce power that is not always steady and can change at any time. Further, the increasing use of DG and the continuous reliance on fossil fuel-based generation have led to the creation of hybrid power systems (PS). These systems try to use more than one source of energy to make the economy and the environment more sustainable. But the uncertainty that comes with RESs has a big impact on the power network’s overall reliability, power quality, and system robustness.Tidal energy has recently become a promising and long-lasting source of energy because to the use of tidal power systems^1,2^.

There are several things that can make the tidal system unstable, such as climate change causing sea levels to rise, which makes tidal flooding more common and worse. These kinds of alterations could disrupt tidal currents, sediment movement, and water quality, which could have a negative effect on the health and stability of coastal and marine habitats^3,4^.

To solve these problems, tidal power plants are using more and more hybridization and auxiliary energy integration solutions, especially in coastal areas. In addition, there has been a lot of research into how to connect tidal energy facilities to the grid in recent years. Doubly fed induction generators (DFIGs) and direct-drive permanent magnet synchronous generators (DDPMSGs) are two types of generators that are becoming more popular in both wind and tidal energy applications because they are very efficient, can be used in a variety of ways, and can be adapted to different situations^5^. Therefore, it is important to look at how these generator setups affect the overall stability of the system^6^. Most DFIG-based turbines have a back-to-back converter in the rotor circuit, which lets them regulate the turbine separately as it starts up and runs^7^. But because DFIG systems have gearboxes, they often have more mechanical losses and maintenance issues unlike DDPMSGs.

In tidal turbines that use DFIG, the rotor current is controlled by changing the converter output voltage^8,9^. In tidal-based hybrid power systems, stability is a major issue because the load changes a lot and renewable supplies are not always reliable^10^.These uncertainties can lead to big changes in voltage and other system parameters that aren’t wanted(11).

UPFCs are often used to control the flow of power and make systems more stable using a variety of control methods^11^. This study advocates for the implementation of a UPFC to enhance voltage stability via reactive power compensation in a DDPMSG-based hybrid power system^12^.

Here intelligent optimization strategies are consistently recommended for achieving optimal design of controllers^13,14^. These methods’ drawbacks include the requirement for accurately configuring algorithm-specific parameters and a high volume of iterations. Nevertheless, these meta-heuristic (M-H) approaches^15,16^ offer two primary benefits. Firstly, they feature an effective information-sharing mechanism that can accelerate convergence under specific circumstances. Secondly, they exhibit a reduced likelihood of becoming stuck in local modes. Utilization of evolutionary and metaheuristic algorithms, specifically DEA and FA^17–19^, as potential ways in addressing the aforementioned issue is noticed in several literatures. In this manuscript, we introduce an innovative amalgamated population-centric global optimization technique, termed the hybrid FA, which amalgamates the strengths of both the FA and DEA. FA and DEA are concurrently applied to foster knowledge dissemination within the population, thereby augmenting the search efficacy. The results produced by these algorithms have been compared to traditional optimization techniques^20,21^. The validation of the control approach is implemented utilizing a real-time approach^22–24^ known as OPAL-RT 5142. The efficacy of the hybrid FA-optimized PID controller is exemplified through a comparative analysis of its simulation outcomes with those of the FA and DEA-assisted PID controller in a real-time scenario.

## Mathematical modeling of the system

**Fig. 1:**
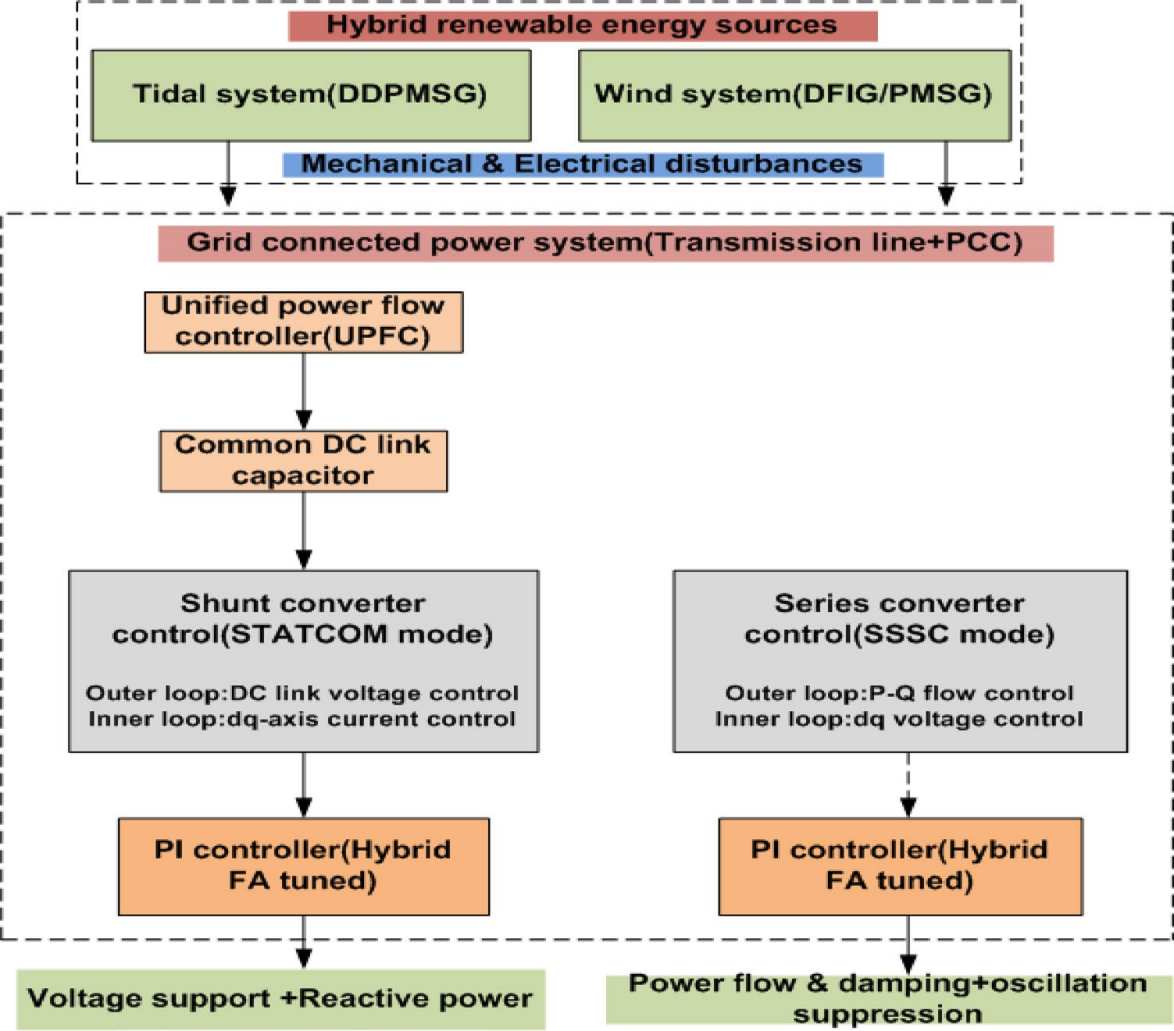
Block diagram of control architecture.

The process of analyzing a tidal-based hybrid PS for small signal analysis entails the simplification of its dynamics in order to examine its response to minor disturbances or fluctuations in operational parameters. Tidal-based hybrid PSs commonly integrate tidal energy with other forms of renewable energy, such as solar or wind, in conjunction with energy storage components Fig. 1. The proposed tidal system comprises a tidal turbine equipped with either DFIG based generator or direct drive PMSG, which is connected to a diesel generator with a SG configuration Fig. 3(a-c) [25]. The DG has been equipped with an excitation system of IEEE Type-1, as shown in Fig. 5. The hybrid PS operating on Tidal energy has experienced a gradual change in the applied load, resulting in slight modifications to system parameters and impacting the management of reactive power across the whole system. A slight deviation into the actual energy magnitude has an impact upon the system frequency, whereas the fluctuation in the reactive power influences the terminal voltage of hybrid PS^26^.

**Fig. 2:**
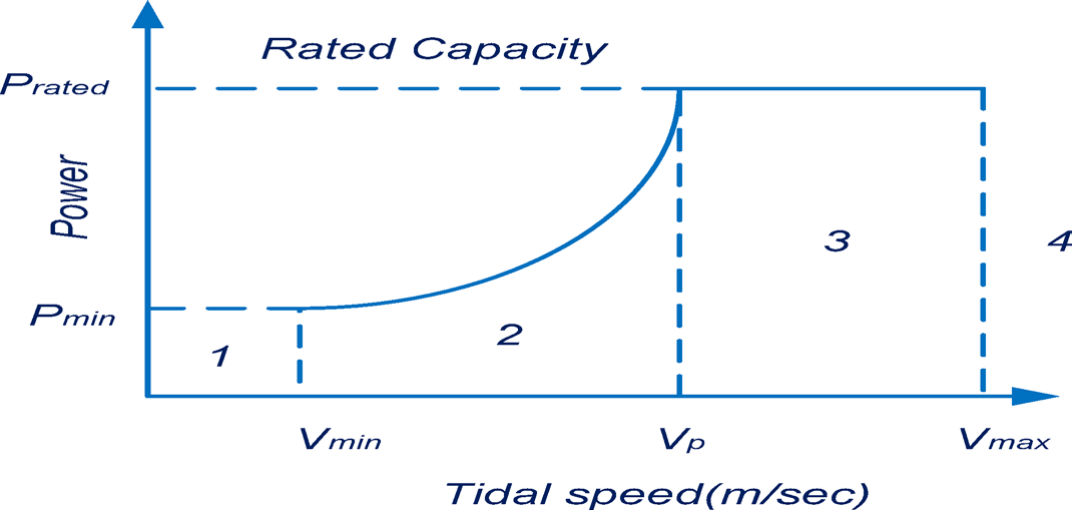
Relationship between Power and Velocity in a standard Tidal power facility.

**Fig. 3:**
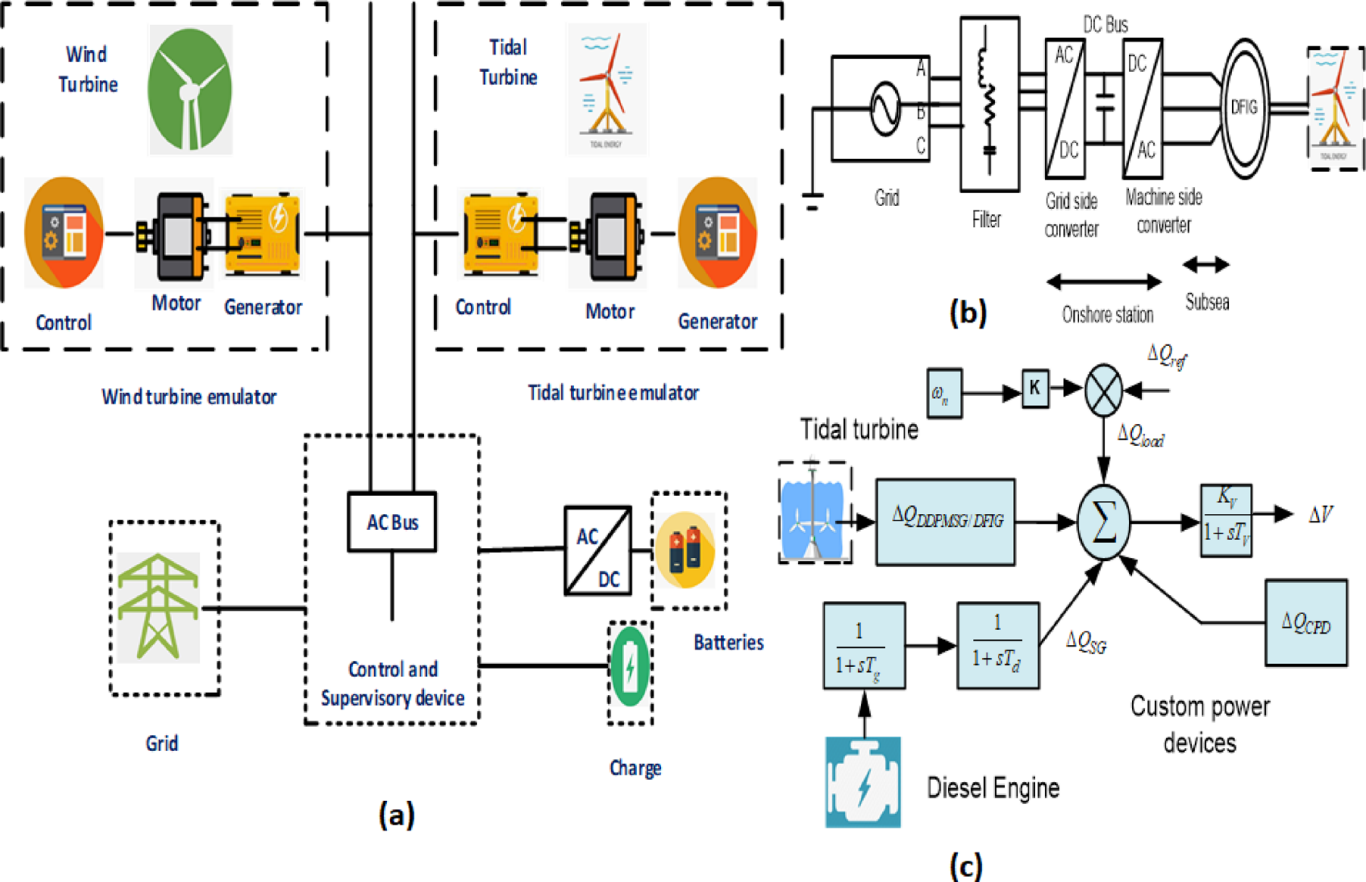
(**a**). Tidal energy based hybrid PS set up, **(b)**Introduction to the Experimental Setup for Tidal Energy, (**c**) Reactive power management of a Tidal hybrid PS.

**Fig. 4:**
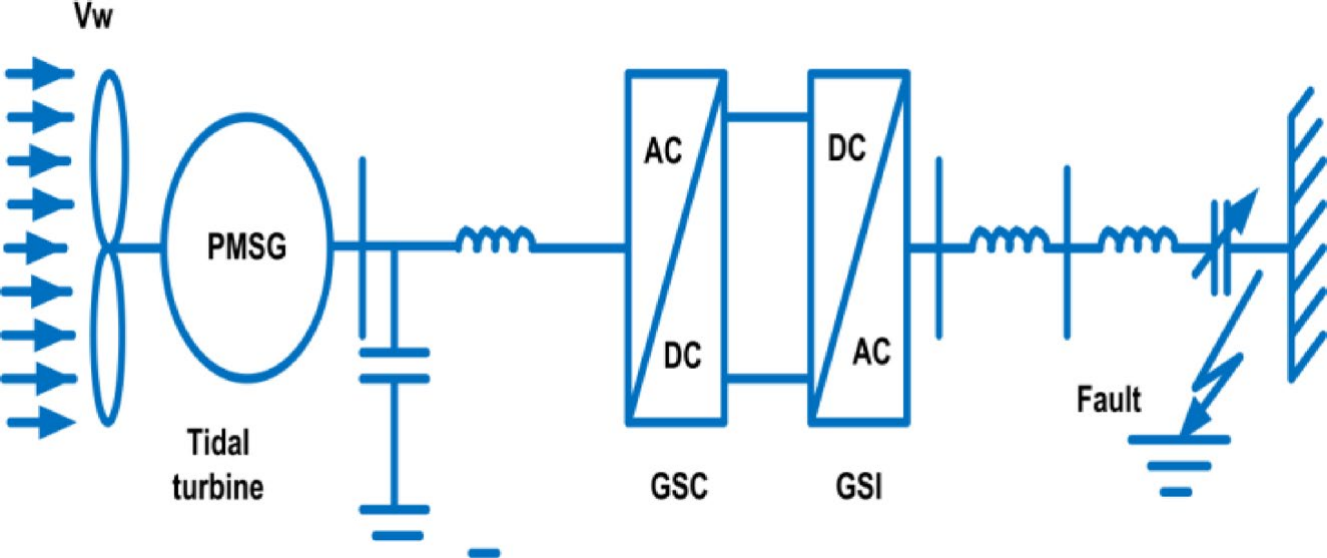
Tidal Turbine model.

Understanding the relationship among power as well as tidal water velocity is vital for identifying the suitable control method, optimization tactics, and constraints. A power graph can be generated to approximate the available energy which is harvested from the incoming tide. Figure 1. displays an optimized performance graph for a tidal turbine, demonstrating the specified operational constraints such as the entry and exit speeds. Furthermore, it can be ensured that the power grade in the interval stays higher than the minor requirement, thus safeguarding the overall structural strength^27^. The manufacturer’s rated power reflects a balance between energy efficiency and economic considerations. The reliability of tidal power generation technologies could be reinforced with the increasing volume of studies in the domain of tidal energy systems Fig. 2(a), Figs. 3 and 4.

The proposed tidal hybrid generation system comprises of a direct-drive permanent magnet synchronous generator(DDPMSG), a back-to-back voltage source converter(VSC) with grid side and generator side converter, a DC-link capacitor, and a local hybrid energy storage element for power smoothing^28^.The system dynamics are represented through an integrated electrochemical and control oriented model to facilitate both time domain and small signal stability analysis.

### Hydrodynamic model of the tidal turbine

The mechanical power extracted from the tidal flow is expressed as

1 $${P}_{t}=\frac{1}{2}{\rho}_{A{C}_{p}}\left(\lambda,\beta\right){v}_{t}^{3}$$

Where $\rho$ stands for sea water density.

$A=\pi{R}^{2}$ is the turbine swept area.

${v}_{t}$ indicates the tidal current velocity(1–3 m/sec typical range).

${C}_{p}\left(\lambda,\beta\right)$ is the power coefficient dependent on tip speed ratio $\lambda={\omega}_{t}R/{v}_{t}$ and blade pitch angle $\beta$.

The turbine mechanical torque is

2 $${T}_{t}=\frac{{P}_{t}}{{\omega}_{t}}=\frac{1}{2\rho AR{C}_{P}\left(\lambda,\beta\right){v}_{t}^{3}}{\omega}_{t}$$

For variable speed operation, maximum power point tracking(MPPT) maintains the optimal tip-speed ratio ${\lambda}_{opt}$^29^, so

3 $${T}_{t}^{*}={K}_{opt}{\omega}_{t}^{2},{K}_{opt}=\frac{1}{2}\rho A{R}^{5}\frac{{C}_{P},max}{{\lambda}_{opt}^{3}}$$

### Electric model of the Direct-Drive PMSG

In the rotor reference dq frame, the voltage equations of non silient(surface mounted) PMSG are

4 $${v}_{d}={R}_{s}{i}_{d}-{\omega}_{e}{L}_{q}{i}_{q}$$

5 $${v}_{q}={R}_{s}{i}_{q}+{\omega}_{e}{L}_{d}{i}_{d}+{\omega}_{e}{\psi}_{f}$$

Where ${v}_{d,}{v}_{q}$ are stator voltages.

${i}_{d},{i}_{q}$ are stator currents.

${R}_{s}$ is the stator resistance.

${L}_{d},{L}_{q}$ are the dq axis inductances.

${\psi}_{f}$ is the rotor flux linkage.

${\omega}_{e}=p{\omega}_{r}$ is the electrical angular frequency with $p$ denoting number of pole pairs^30^.

The electromagnetic torque is

6 $${T}_{e}=\frac{3}{2}p\left[{\psi}_{f}{i}_{q}+\left({L}_{d}-{L}_{q}\right){i}_{d}{i}_{q}\right]$$

For a surface mounted PMSG, 

7 $${L}_{d}={L}_{q}={L}_{s},\, {\rm hence}\,\, {T}_{e}=\frac{3}{2}p{\psi}_{f}{i}_{q}$$

The mechanical equation of motion is.

$J\frac{d{\omega}_{r}}{dt}={T}_{t}-{T}_{e}-{B\omega}_{r}$, where $J$ is the total inertia and $B$ is the viscous friction coefficient.

### Converter and DC-link model

The back-to-back converter comprises a generator side converter(GSC) and grid side converter(GRC) connected via a DC link capacitor ${C}_{dc}$.

The DC-link voltage dynamic is governed by ${C}_{dc}\frac{d{V}_{dc}}{dt}={i}_{dc}.GSC-{i}_{dc},GRC$.

Where ${i}_{dc}.GSC,{i}_{dc},GRC$ are the DC currents injected or absorbed by the generator side and grid side converters respectively.

Each converter phase current(in dq form) obeys

8 $${L}_{f}\frac{{di}_{gd}}{dt}=-{R}_{f}{i}_{gd}+{\omega}_{s}{L}_{f}{i}_{gq}+{v}_{cd}-{v}_{gd}$$

9 $${L}_{f}\frac{{di}_{gq}}{dt}=-{R}_{f}{i}_{gq}-{\omega}_{s}{L}_{f}{i}_{gd}+{v}_{cq}-{v}_{gq}$$

${v}_{cd},{v}_{cq}$ are converter output voltages,${v}_{gd,}{v}_{gq}$ are grid voltages in dq frames, and ${R}_{f},{L}_{f}$ represent the filter impedance.

### Control sub system design

(a)Generator side converter(GSC)-regulates the PMSG speed and extracts maximum power using an outer loop speed controller and an inner loop controller.The control laws are

10 $${i}_{q}^{*}=\frac{2}{3p{\psi}_{f}}\left({T}_{t}^{*}+{K}_{\omega}\left({\omega}_{t}^{*}-{\omega}_{t}\right)\right)$$

11 $${v}_{d,q}^{*}={R}_{s}{i}_{d,q}+{L}_{S}\frac{{di}_{d,q}}{dt}\mp{\omega}_{e}{L}_{q,d}{i}_{q,d}+{v}_{d,q}^{PI}$$

(b)Grid side converter(GSC)-Maintains DC-link voltage and regulates reactive power at the point of common coupling(PCC).The DC-link PI controller output defines the reference current ${i}_{gd}^{*},$while ${i}_{gq}^{*}$ is set by the reactive power reference ${Q}^{*}$

12 $${v}_{gd}^{*}={v}_{gd}+{R}_{f}{i}_{gd}+{L}_{f}\frac{{di}_{gd}}{dt}-{\omega}_{s}{L}_{f}{i}_{gq}$$

13 $${v}_{gq}^{*}={v}_{gq}+{R}_{f}{i}_{gq}+{L}_{f}\frac{{di}_{gq}}{dt}+{\omega}_{s}{L}_{f}{i}_{gd}$$

Each control loop is linearized around the nominal operating point to form the small signal model for eigenvalue analysis.

### Linearization and state space representation

Let the nonlinear model be written as:

$$\dot{x}=f\left(x,u\right),y=g\left(x,u\right)$$

Where the state vector is $x={\left[{i}_{d},{i}_{q},{i}_{gd},{i}_{gq},{\omega}_{r},{V}_{dc}\right]}^{T}$.

And control inputs are $u={\left[{v}_{cd},{v}_{cq},{v}_{gd},{v}_{gq}\right]}^{T}$.

Linearization about a steady state operating point$\left({x}_{0},{y}_{0}\right)$ yields

$$\varDelta\dot{x}=A\varDelta x+B\varDelta u,\varDelta y=C\varDelta x+D\varDelta u$$

Where $$A={\left.\frac{\partial f}{\partial x}\right|}_{{x}_{0},{u}_{0}},B={\left.\frac{\partial f}{\partial u}\right|}_{{x}_{0},{u}_{0}}$$.

Reactive power balanced equation of this Tidal based hybrid PS Fig. 2(b)

14 $${Q}_{SG}+{Q}_{UPFC}={Q}_{L}+{Q}_{DFIG/DDPMSG}$$

With load variation $\varDelta{Q}_{L}$

15 $$del{Q}_{SG}+del{Q}_{UPFC}=del{Q}_{L}+del{Q}_{DFIG/DDPMSG}$$

16 $$del{Q}_{SG}+del{Q}_{UPFC}-del{Q}_{L}-del{Q}_{\frac{DFIG}{DDPMSG}}=0$$

is the reactive power balance equation of the system.

The modified equation is

17 $$\varDelta V\left(S\right)=\frac{{K}_{V}}{1+s{T}_{V}}\left[\varDelta{Q}_{SG}\left(S\right)+\varDelta{Q}_{COM}\left(S\right)-\varDelta{Q}_{L}\left(S\right)-\varDelta{Q}_{DFIG/DDPMSG}\left(S\right)\right]$$

Where $${T}_{v}=\frac{2{H}_{r}}{{D}_{v}}{v}^{0}\&{K}_{v}=\frac{1}{{D}_{v}}.$$

The provided figures, specifically Figure 3(c), illustrates the customary approach to small signal modeling of the Tidal-based hybrid PS. This system composes of a Tidal turbine integrated with either a DFIG or a DDPMSG, as well as a diesel engine coupled with a synchronous generator[Figure.4]. These components are interconnected with electrical loads. Consistency and responsive power control can be effectively accomplished by utilizing custom power devices such as the UPFC.

The equation for the small signal modeling of a synchronous generator is as follows:

${Q}_{SG}=\frac{\left({E}_{q}^{{\prime}}Vcos\delta-{V}^{2}\right)}{{X}^{{\prime}}d}$ in transient condition.

For an incremental variation

18 $$\varDelta{Q}_{SG}=\frac{Vcos\delta}{{X}^{{\prime}}d\varDelta{E}^{{\prime}}}+\frac{{E}^{{\prime}}qcos\delta-2V}{{X}^{{\prime}}d\varDelta V}$$

With the Laplace Equation the relation is

$$\varDelta{Q}_{SG}\left(S\right)={K}_{a}\varDelta{E}^{{\prime}}q\left(S\right)+{K}_{b}\varDelta V\left(S\right)$$

$${K}_{a}=\frac{Vcos\delta}{{X}^{{\prime}}d}\&{K}_{b}=\frac{{E}^{{\prime}}qcos\delta-2V}{{X}^{{\prime}}d}$$

**Fig. 5:**
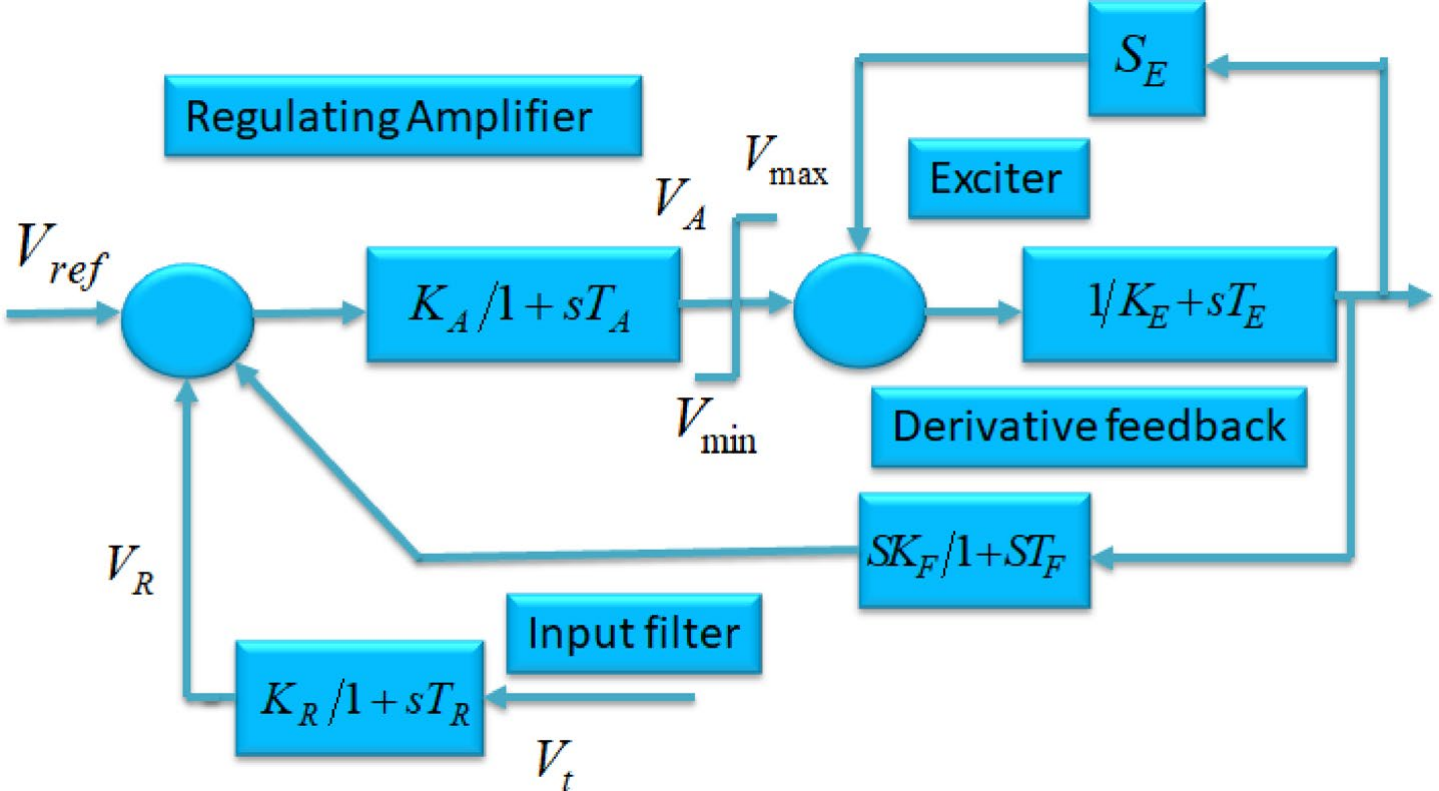
IEEE specified AVR Model (Type 1).

The regulation mechanism has typically been depicted by employing a high-gain AVR with a single time constant. Among the various AVR configurations available, this integrated hybrid system employs the IEEE Type I AVR model, as shown in Fig. 5.

#### A. DFIG based tidal turbine

**Fig. 6:**
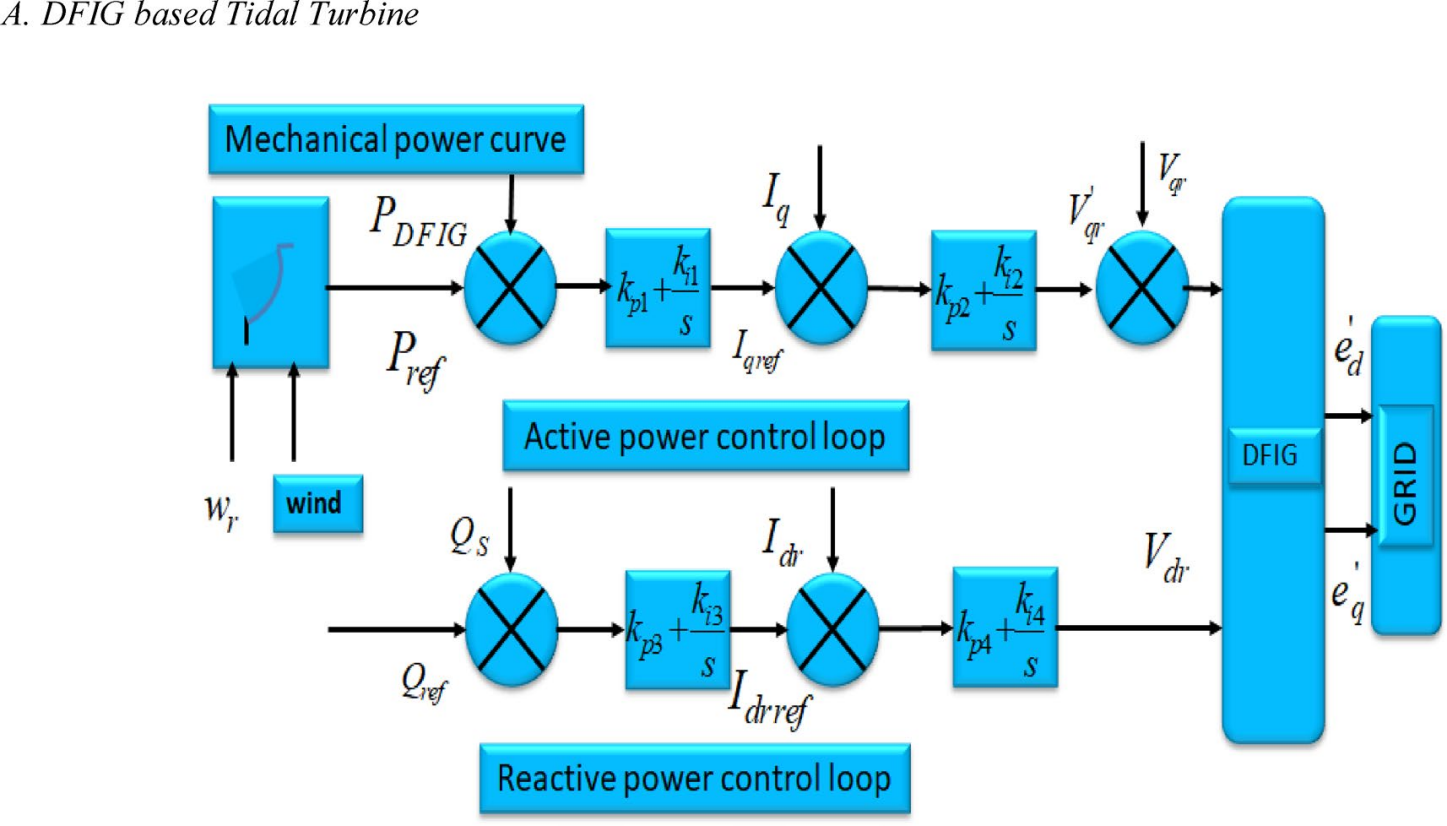
The control strategy implemented for the PI Controller on the rotor side of DFIG.

The block diagram of a single line DFIG is depicted in Fig. 6. In this figure, the supply section of the DFIG is responsible for maintaining a constant DC voltage, disregarding the direction of power transfer in the rotor. The management of the generator is determined by the location of the stator-flux vector within the synchronously rotating dq-axis. The management of both active as well as reactive power is accomplished by implementing Iqr and the Idr control in the converter located near the rotor section. The diesel engine within the hybrid PS effectively regulates both active and reactive power during instances of elevated and fluctuating loads. The management of reactive output energy has been accomplished by utilizing independent proportional-integral (PI) controllers. The external loop compares the DFIG voltage with a reference voltage.

19 $${Q}_{DFIG}=\frac{{L}_{m}}{{L}_{ss}}{V}_{1}{I}_{dr}-\frac{{V}_{1}^{2}}{{w}_{s}{L}_{ss}}$$

The reactive power associated by DFIG, as illustrated in Fig. 5, is

20 $$\varDelta{Q}_{DFIG}\left(s\right)={K}_{f}\varDelta{I}_{dr}\left(s\right)+{K}_{e}\varDelta V\left(s\right)$$

Where $${K}_{f}=\frac{{L}_{m}{V}_{1}}{{L}_{SS}}\&{K}_{e}=\frac{{L}_{m}{I}_{dr}}{{L}_{ss}}-\frac{2{V}_{1}}{{w}_{s}{L}_{SS}}$$.

21 $$\varDelta{I}_{dr}^{ref}=\left({K}_{P}+\frac{{K}_{I}}{S}\right)\left[{\varDelta V}^{ref}\left(s\right)-\varDelta V\left(s\right)\right]$$

The responsive internal loop is modeled like

$${\varDelta I}_{dr}=\frac{1}{\left(1+{}_{4}{}^{{t}_{s}}s\right){\varDelta I}_{dr}^{ref}}$$

$$\underset{\_}{X}=\left[del{I}_{dr}^{ref},del{I}_{dr},delV,del{E}_{fd},del{V}_{a},del{V}_{f},del{E}_{q}^{{\prime}}\right]$$

$$\underset{\_}{u}=\left[del{V}_{ref}\right]$$

$$\underset{\_}{w}=\left[del{Q}_{L}\right]$$

#### B. DDPMSG based Tidal turbine

**Fig. 7:**
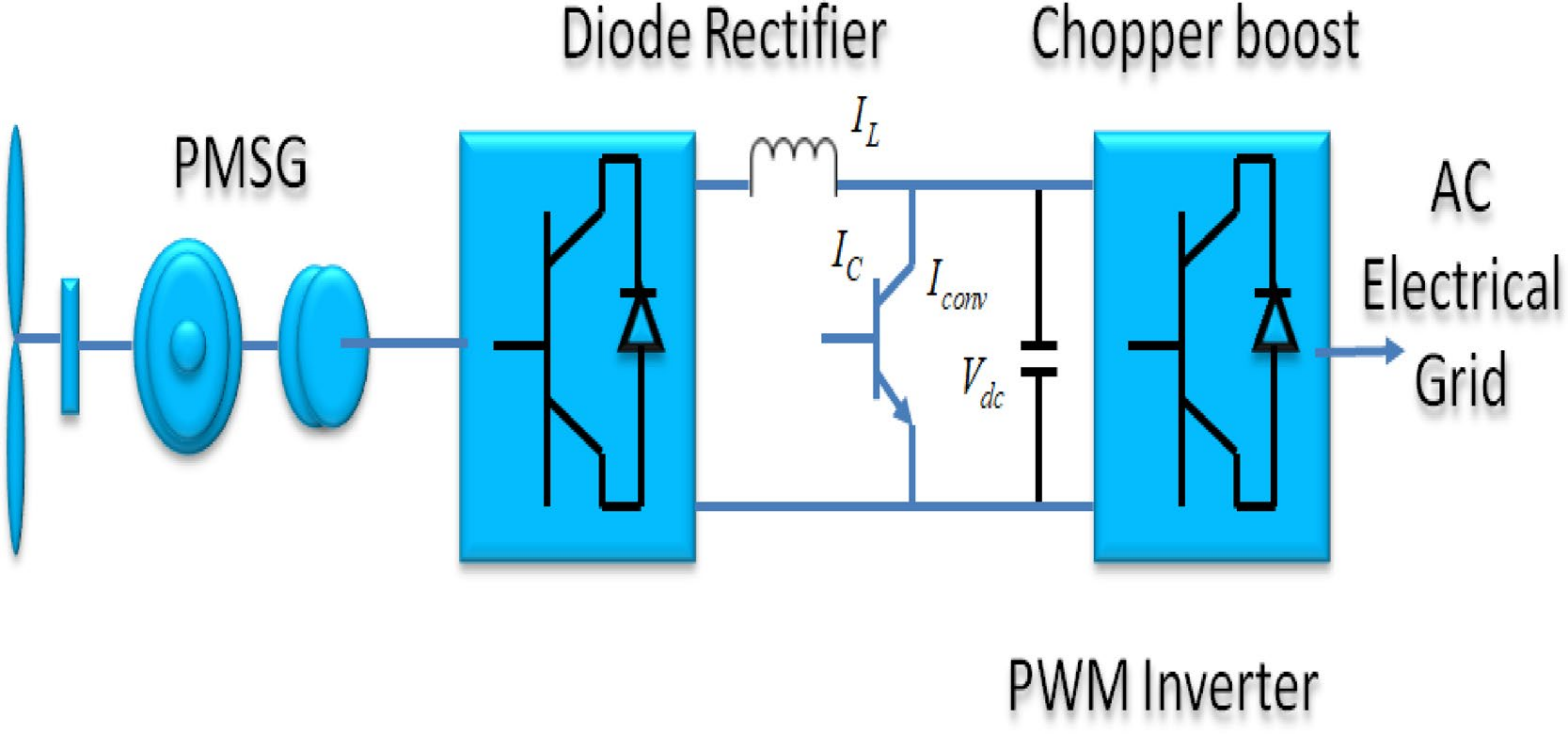
Arrangement of a generator based on PMSG.

The DDPMSG uses a rectifier as the converter on the generator-side, while the converter on the grid section remains a pulse width modulation (PWM) inverter Fig. 7. These are utilized achieving the targeted DC bus voltage. Additionally, controlling the power factor of the grid portion is accomplished using a separated d-q-based vector control method. In addition, the DC-link functions to provide isolation between the converters’ operations.

The DDPMSG operates similarly to a synchronous generator with a rotor that has a uniform flux linkage. The conventional formula thorough DDPMSG is specified as

22 $${L}_{S}\frac{d{i}_{ds}}{dt}=-{v}_{ds}-{R}_{s}{i}_{ds}+{L}_{s}w{i}_{qs}$$

23 $${L}_{S}\frac{d{i}_{qs}}{dt}=-{v}_{qs}-{R}_{s}{i}_{qs}+{L}_{s}w{i}_{ds}+\omega\psi$$

By orienting the d-axis of the d-q reference frame in accordance with the flux linkage. The variable ${V}_{ds}$ denotes the stator voltage’s direct axis, whilst ${V}_{qs}$ indicates the quadrature axis (q) in stator voltages. In addition to this, the initials ${i}_{ds},{i}_{qs}$ denote the stator currents. Also, ${R_s}$ represents the stator resistance, whereas ${L_s}$ signifies the stator inductance and ω denotes the generator’s velocity.

Hence, Power equations can be formulated as

$${P}_{S}={V}_{ds}{i}_{ds}+{V}_{qs}{i}_{qs}\&{Q}_{S}={V}_{qs}{i}_{ds}-{V}_{ds}{i}_{qs}$$

The converter located on the generator side is tasked with the regulation of the active power generated by the generator. This is achieved through the careful monitoring of the input of torque from the tidal turbine. The primary aim of this system is to reduce power losses in the generator. The regulation of additional active power is achieved by controlling the voltage component in the synchronous reference frame ${V}_{qs}$, while the reduction of power losses in power generators is accomplished by effectively managing the direct current component in the synchronous reference frame ${i}_{ds}$ to approach zero. Reactive power management has been executed utilizing the variable voltage source ${V}_{ds}$.

The converter on the generator side is responsible for regulating the active power of the generator by monitoring the input torque from the Tidal turbine. The fundamental aim is to mitigate power losses within the generator. The regulation of additional active power is achieved by controlling the voltage component in the synchronous reference frame (${V}_{qs}$), while minimizing power losses in power generators is accomplished by effectively managing the direct current component in the synchronous reference frame (${i}_{ds}$) to approach zero. The execution of regulation is conducted employing the variant voltage supply (${V}_{ds}$). Equations depending upon the control strategy have been derived as follows.

$$X=\dot{f}\left[X,Z,u\right]\&Z=\left[g\left[X,u\right]\right]$$

$$X={\left[{\omega}_{r,}{i}_{ds},{i}_{qs},{V}_{dc},{X}_{1},{X}_{2},{X}_{3},{X}_{4},{X}_{5},{X}_{6}\right]}^{T}$$

$$Z={\left[{V}_{ds},{V}_{qs},{V}_{Dg},{V}_{Qg}\right]}^{T}$$

$${U=\left[{V}_{D1},{V}_{Q1},{i}_{Dg},{i}_{Qg}\right]}^{T}$$

The vectors x, z, and u are associated with the state variables.

$$\varDelta\dot{x}$$=$${A}^{{\prime}}delx+{B}^{{\prime}}z+{C}^{{\prime}}u$$&$$\varDelta z={D}^{{\prime}}x+{E}^{{\prime}}u$$.

**Fig. 8:**
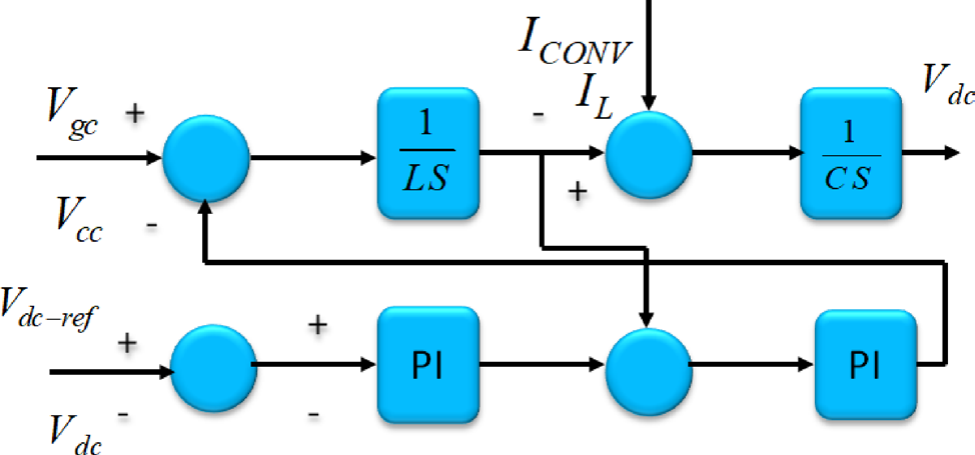
Control circuit concerning the DC-linked voltage.

The transformer positioned on the grid aspect maintains a reliable DC-linked voltage depicted in Fig. 8, as well as impacts the end voltage. Employing reactive power regulation within the Tidal-powered hybrid PS improves the voltage steadiness of the system. In the context of Tidal-driven hybrid PS, for DC-linked and terminal voltages undergo regulation through${V}_{Dg}\&{V}_{Qg}$

$$\begin{aligned}{V}_{Dg}=&{K}_{P5}\left(-{K}_{P3}\varDelta{C}_{DC}+{K}_{i3}{X}_{3}-i{D}_{g}\right){K}_{i5}{X}_{5}+{X}_{C}i{Q}_{g})\&{V}_{Qg}\\&={K}_{P5}\left(-{K}_{P4}\varDelta{V}_{t}+{K}_{i4}{X}_{5}-i{Q}_{g}\right){K}_{i5}{X}_{6}+{X}_{C}{i}_{Dg}\end{aligned}$$

${K}_{P5},{K}_{i5}$highlight the improvements associated with the grid converter, the terminal voltage regulator, and the dc-bus voltage regulation. The matrix A denotes the combined system state matrix in the DDPMSG-based Tidal hybrid PS Fig. 8. A represents the overall matrix in DDPMSG based hybrid PS.

$$\left[\begin{array}{c}\underset{\_}{x}={\left[del{\omega}_{t},del{i}_{ds},del{i}_{qs},del{V}_{dc},delV,del{E}_{fd},del{V}_{a},del{V}_{f},{delE}_{q}^{{\prime}}\right]}^{T}\\\underset{\_}{u}=\left[del{V}_{ref}\right]\\\underset{\_}{w}=\left[del{Q}_{L}\right]\end{array}\right]$$

**Fig. 9:**
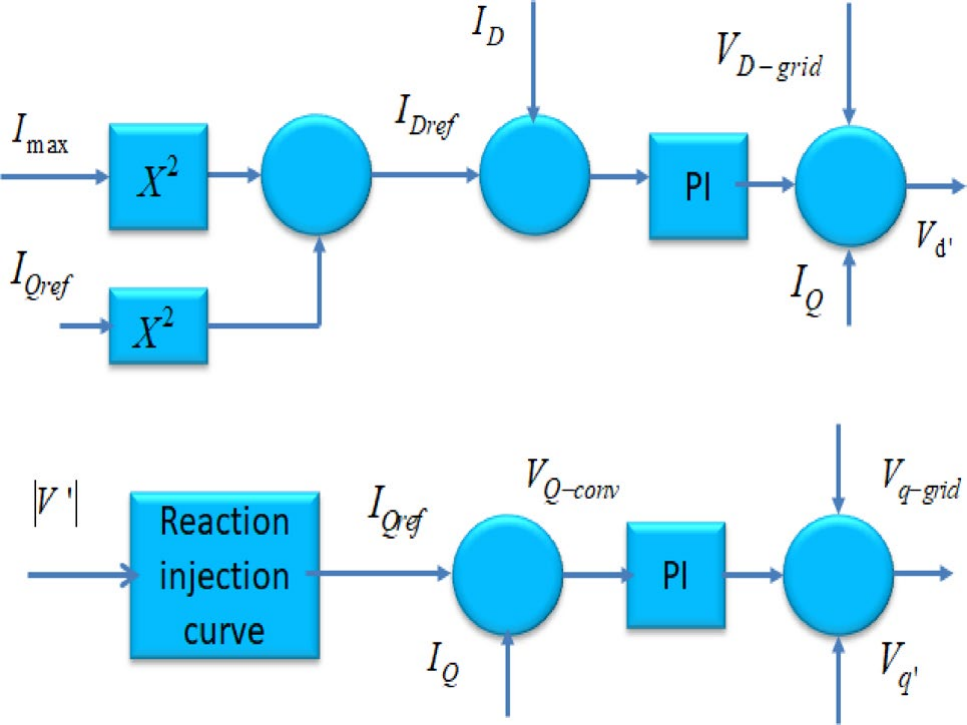
Converter on the grid side of the Tidal-based hybrid PS featuring DDPMSG.

**Fig. 10:**
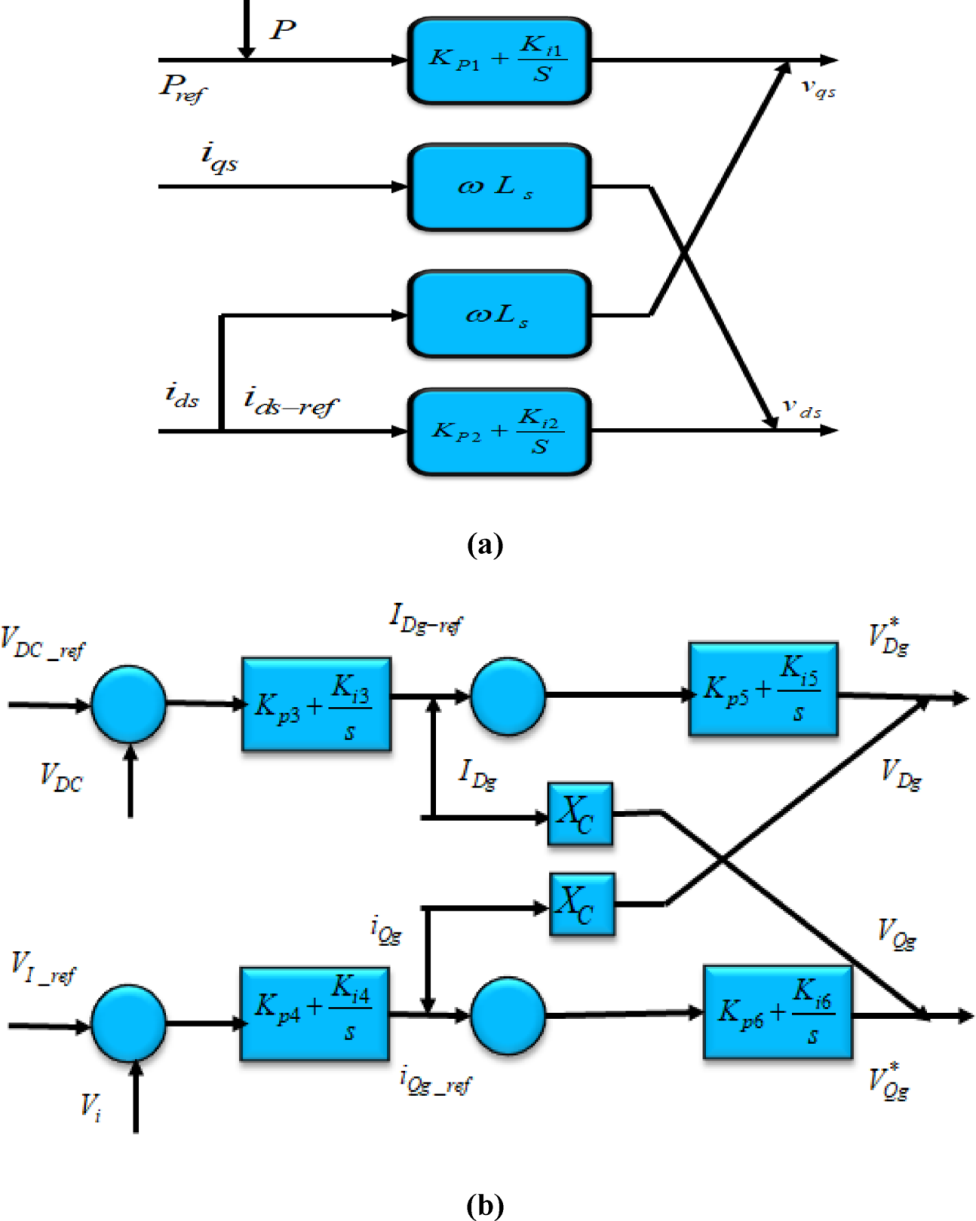
(**a**). Diagram depicting the generator aspect, (**b**). Diagram for side of grid.

In the Tidal-powered hybrid PS with integrated DDPMSG, in the electricity network Figs. 9 and 10(a-b).

The system is modeled as

$$H\frac{d{w}_{t}}{dt}={T}_{M}-{T}_{e}$$

Where,

$${T}_{M}=\frac{0.5\rho\pi{R}^{2}{C}_{P}{V}_{W}^{3}}{{\omega}_{T}}$$

and

24 $${C}_{p}=\frac{1}{2}\left(\frac{R{C}_{f}}{\lambda}-0.022{\beta}^{-2}\right){e}^{-0.255\left(R{C}_{f}/\lambda\right)}$$

Here $${T}_{e}=-p\psi{i}_{qs}$$.

#### C. UPFC controller

The UPFC is a type of FACTS controller that is utilized for reactive power compensation and concurrent enhancement of the voltage. The UPFC is a device that comprises two voltage source inverters, one connected in series and the other in parallel to the electric conduit. The system regulates both reactive power and manages active power through the injection of alternating current voltage into the electric cable.

This study chooses the UPFC because it can manage active power, reactive power, and bus voltage at the same time and separately, which single-function FACTS devices can’t do. In hybrid tidal-wind systems utilizing DDPMSGs, disturbances arising from mechanical intermittency influence both voltage and power flow dynamics. SVC and STATCOM are two examples of devices that predominantly provide shunt-based reactive power compensation and voltage support. TCSC and SSSC are examples of series compensators that largely affect power flow without directly regulating voltage. The UPFC, on the other hand, connects shunt and series converters through a shared DC-link. This makes it possible to coordinate damping augmentation, voltage stability, and power flow management. This ability to regulate many things at once makes UPFC very good at reducing problems with coupled electromechanical and grid-side instabilities in hybrid renewable systems.

#### D. Optimization of UPFC controller

When applying an optimization strategy to a controller, the formulation of the goal function is based on specified requirements and constraints^31^. The goal function plays a crucial role in the adjustment of the controller and is closely associated with the performance index, which encompasses the entirety of the closed-loop system. In this study, performance indices such as the Integral of Time multiplied by Absolute Error (ITAE) have been taken into account, which incorporates the cumulative sum of absolute discrepancies multiplied by duration during a specific interval of duration^32^. The objective function has been formulated as

$$J=IATE=\underset{0}{\overset{\infty}{\int}}\left|\varDelta V\right|tdt$$

Where, $\varDelta V$indicates the system’s voltage deviation. The problem restrictions outlined in this context are contingent upon the control settings specified. The optimization problem can be solved by minimizing the objective function J across a specified range of ${K}_{{I}_{min}}\le{K}_{I}\le{K}_{{I}_{max}}$.

${K}_{{I}_{min}}\&{K}_{{I}_{max}}$ represent the optimal settings of control variables.

#### E. Differential evolution based UPFC controller

The optimisation process involves maintaining a population of potential solutions and producing new solutions based on existing ones using straightforward mathematical formulas. The DEA method possesses the ability to handle non-linearities and has the capability to optimise multi-objective functions.

#### F. Firefly algorithm

**Fig. 11:**
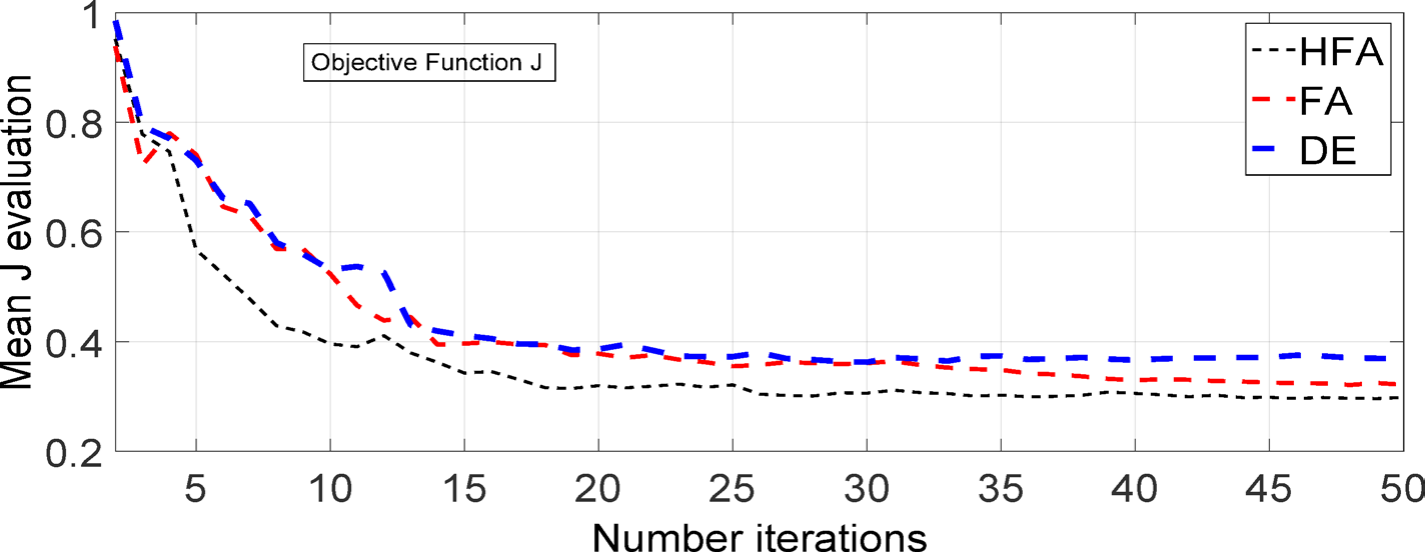
Convergence Curve for DEA, FA and hybrid FA.

The application of the FA has been provided by the authors, as depicted in Fig. 11. A comparison has also been conducted, incorporating several different algorithms such as DEA. As previously stated, FA demonstrates competence as an intermittent search technique. The phenomenon is predicated upon the intermittent flashing behaviour shown by fireflies. Primarily, FA primarily adheres to three fundamental principles: Given that all fireflies are unisex, they possess the ability to attract one another based on their sexual characteristics. There exists a positive correlation between attractiveness and brightness, whereby proximity enhances brightness while distance diminishes it. Therefore, according to the idea, fireflies with lower brightness levels are able to relocate towards fireflies with higher brightness levels. If the level of brightness is consistent, the entities have the ability to travel in a random manner. The evaluation of the objective function will be based on the brightness of the firefly. As previously stated, there exists a direct correlation between brightness and beauty, which may be operationally described as (13).

25 $$I\left(r\right)={I}_{O}{e}^{-\gamma}r$$

The variables I, γ, and r represent the parameters of attraction, light-absorbing constant as well as distance, respectively. The aesthetic appeal of the firefly can be quantified as (14).

26 $$\beta={\beta}_{0}{e}^{-\gamma{r}^{2}}$$

The phenomenon of firefly relocation being influenced by the presence of brighter fireflies can be approximated as

27 $${x}_{i}^{t+1}={x}_{i}^{t}+\beta exp\left[-\gamma{r}_{ij}^{2}\right]\left({x}_{j}^{t}-{x}_{i}^{t}\right)+{\mathfrak{R}}_{t}{\epsilon}_{t}$$

#### G. Hybrid firefly algorithm

This work presents a novel hybrid method that integrates the strengths of both the FA and the DEA. Previous research and literature have shown that the FA has the ability to automatically divide the population into subgroups based on the attraction mechanism, which is determined by the changing of the light intensity. Further, one of these variants of the FA is capable of avoiding local minima by utilizing long-distance mobility through Lévy flight. The aforementioned benefits indicate that FA have the ability to effectively engage in both exploration and diversification. Moreover, from a technical standpoint, it can be argued that the utilization of the mutation operator and crossover operator in DE contributes to its ability to effectively mix individuals within the population. Consequently, this leads to an enhanced variety within the population.


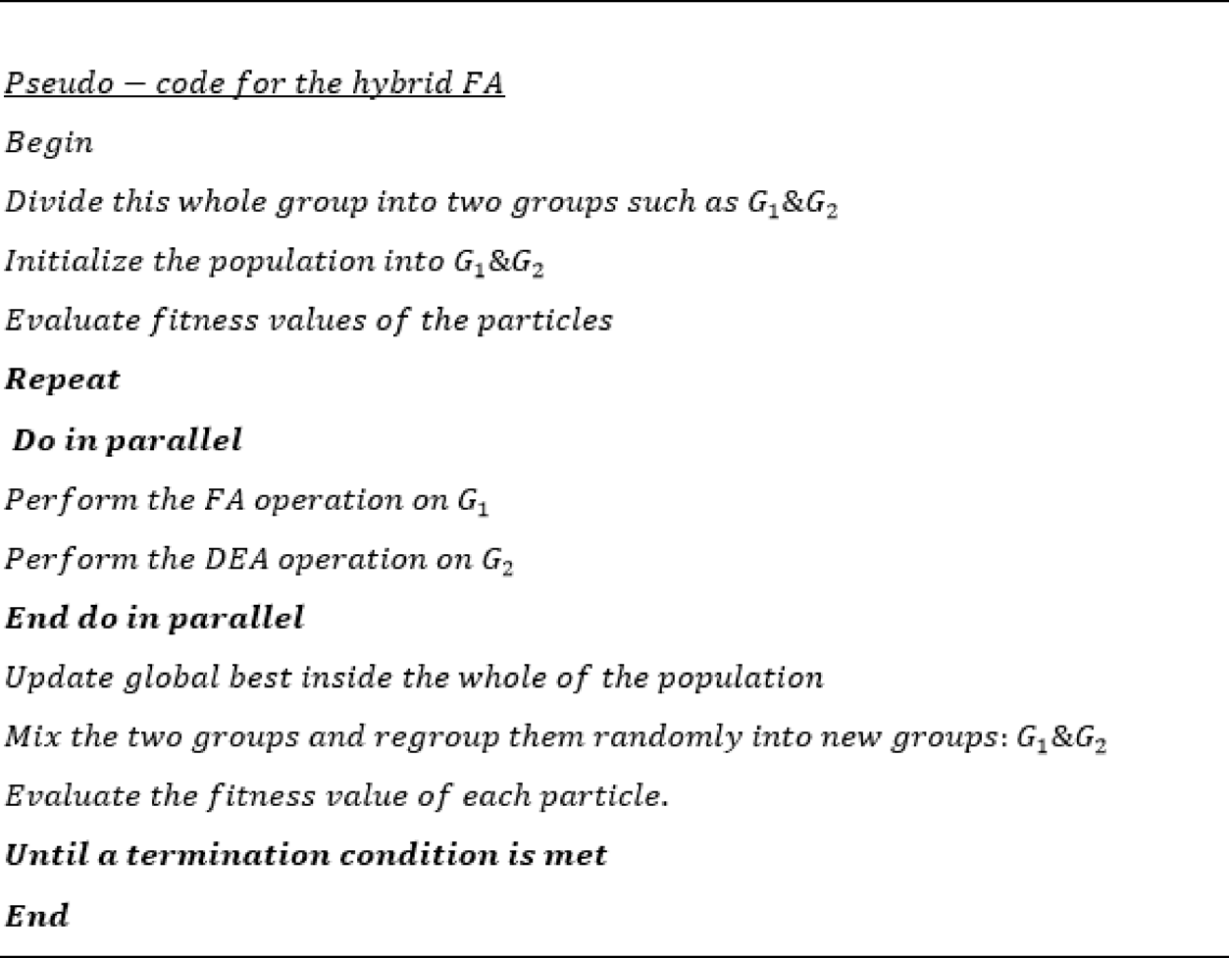


Simultaneously, DEA has the capability to do local search when it nears local optimal solutions. This advantage can be leveraged to enhance the algorithm’s capabilities for both exploitation and exploration. Furthermore, the process of refreshing the current top performer globally best within the entire population guarantees that solutions have the potential to converge towards the optimal solution. Additionally, the process of diversification, achieved through mixing as well as regrouping the entire population, and enabling the entire search algorithm to avoid getting trapped in local optima and potentially enhances the diversity of solutions. It is noteworthy to mention that the process of mixing and regrouping individual location information is limited to the data collected after the primary iteration of concurrent FA as well as DEA processes. This approach does not involve producing novel placements through random movements or alternative operators. The primary advantage of employing a mixing as well as regrouping technique is to ensure that the search process concentrates on the present locations inside the prospective areas identified in the preceding phase, rather than searching less favorable sections of the search space.

While the specific computational intricacies might vary based on the implementation’s structure, the difficulties of the three M-H algorithms employed in this study can be readily approximated. In the context of FA, the time complexity can be expressed as $o\left({n}^{2}t\right)$, where n represents the size of the population and t stands for the iterations. This complexity arises due to the presence of two nested loops that iterate through the population.

The complexity of DEA is $$o\left(nt\right)$$. Hence, in this particular scenario, the temporal complexity of our suggested hybrid FA may be expressed as $$o\left({n}^{2}t/4+\raisebox{1ex}{$nt$}\!\left/\!\raisebox{-1ex}{$2$}\right.\right)$$ due to the fact that each of the components (either FA or DEA) just utilizes half of these populations. Given the small value of n (specifically, *n* = 20 or 40) and the big value of t (specifically, t = 2000), the computational expense is very low due to the linear algorithmic complexity with respect to t. The primary computational expense will arise from the assessments of objective functions as shown in Table 1.

**Table 1:** Adjustment configuration in the tidal-powered hybrid PS for various regulators.

TIDAL based hybrid PS	CONSTANT SLIP		VARIABLE SLIP	
	${K}_{P}$	${K}_{I}$	${K}_{P}$	${K}_{I}$
DDPMSG (optimized)	34	5600	29	5661
DDPMSG (no controller)	46	7600	44	7791
DFIG	71	13,500	60	13,120

Benchmark functions play a crucial role in the assessment of novel algorithms and their attributes, encompassing precision, convergence rate, robustness, and overall performance. In order to assess the efficacy of the proposed algorithm after comparing it to other established algorithms, we employ a collection of 13 standardized benchmark functions. These benchmarks have been deliberately selected to encompass a wide array of attributes. In a theoretical context, it is possible that the utilization of a limited set of benchmark functions could introduce bias in the experimental results. This bias arises from the restricted diversity of the problem objective landscape. Consequently, drawing persuasive conclusions becomes exceedingly challenging in such circumstances.

Hence, we have selected test functions that are predicated on their distinct qualities, modality, and other properties, in order to offer a diverse range of functions with varying levels of difficulty. The test functions employed in this study were identical to those utilized in previous studies[Table 2].

**Table 2:** Results of the DEA and FA and hybrid FA algorithms in comparison to other optimization tools.

Algorithm		Kp		Kd	Mp	Ess	tr	ts	J(pu)
CNC	worst	8.61	3.47	1.89	0.343	0.004	0.03	0.18	8.805
	mean	6.31	2.34	1.29	0.297	0.007	0.03	0.27	7.768
	Best	5.66	0.82	1.95	0.117	0.009	0.11	0.36	6.765
ABC	worst	1.96	0.49	0.23	0.011	0.011	0.17	0.22	5.212
	mean	1.96	0.49	0.23	0.010	0.011	0.18	0.19	5.414
	Best	1.95	0.48	0.23	0.010	0.010	0.19	0.56	5.113
PSO	worst	2.28	0.68	1.55	0.015	0.004	0.17	0.22	5.745
	mean	2.02	0.52	0.24	0.013	0.009	0.15	0.21	5.202
	Best	1.87	0.45	0.23	0.012	0.018	0.16	0.27	5.113
DEA	worst	1.65	3.69	0.32	0.467	0.005	0.03	0.27	9.878
	mean	2.50	1.08	0.40	0.033	0.016	0.10	0.23	6.277
	Best	1.91	0.47	0.22	0.010	0.011	0.18	0.22	5.344
FA	worst	2.27	0.05	0.21	0.012	0.005	0.31	0.20	5.16
	mean	1.92	0.04	0.20	0.010	0.006	0.06	0.18	5.12
	Best	1.66	0.04	0.18	0.009	0.008	0.10	0.18	5.10
Hybrid FA	worst	2.03	0.03	0.18	0.011	0.007	0.3	0.17	4.342
	mean	1.98	0.03	0.16	0.010	0.008	0.05	0.17	4.221
	Best	1.87	0.02	0.15	0.006	0.008	0.09	0.16	4.011

**Fig. 12:**
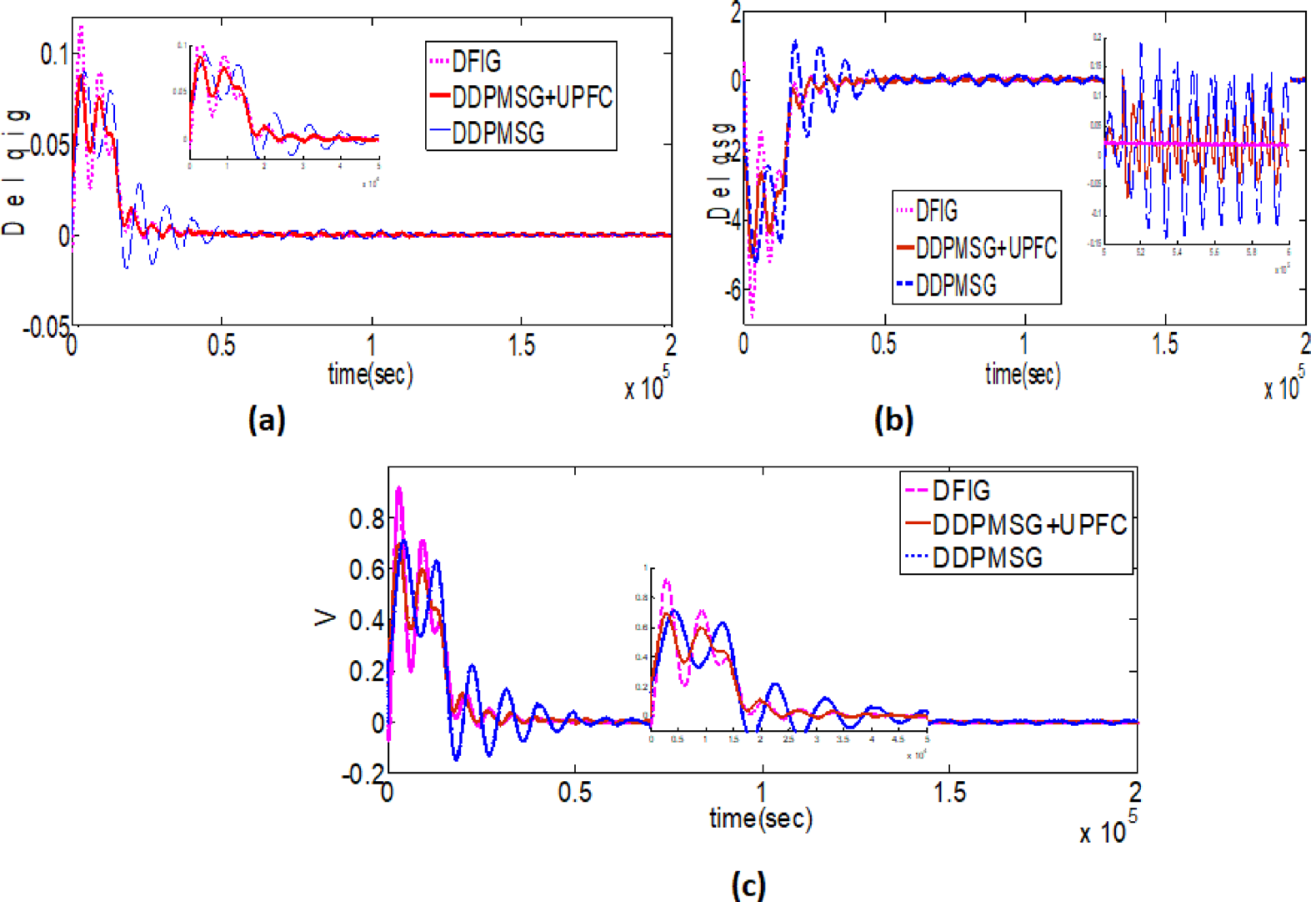
(**a-c**). Comparison outcomes between UPFC-based DDPMSG and DFIG.

Figure 11 illustrates the convergence graph (performance metric versus quantity of performance assessments). Each algorithm has been evaluated using a specific quantity of performance assessments. The selection of these fitness evaluations may be undertaken with the objective of determining the algorithm that yields the highest fitness value. Subsequent to this juncture, the algorithms exhibit an inability to further diminish. The performance trend plots presented in Fig. 12 provide evidence that the Firefly method outperforms two other algorithms, namely DEA as well as PSO, considering speed of convergence as well as also minimal fitness level. A greater pace of convergence suggests achieving a reduced fitness value in a specific duration. The mathematical formula can be utilized to compute the rate of convergence.

$$\frac{\sum_{n=1}^{N}\left({f}_{n+1}-{f}_{n}\right)}{N}$$

${f_n}$ represents the assessment fitness for the n-th trial, with N indicating the overall amount of assessments.

## Results and discussion

**Fig. 13:**
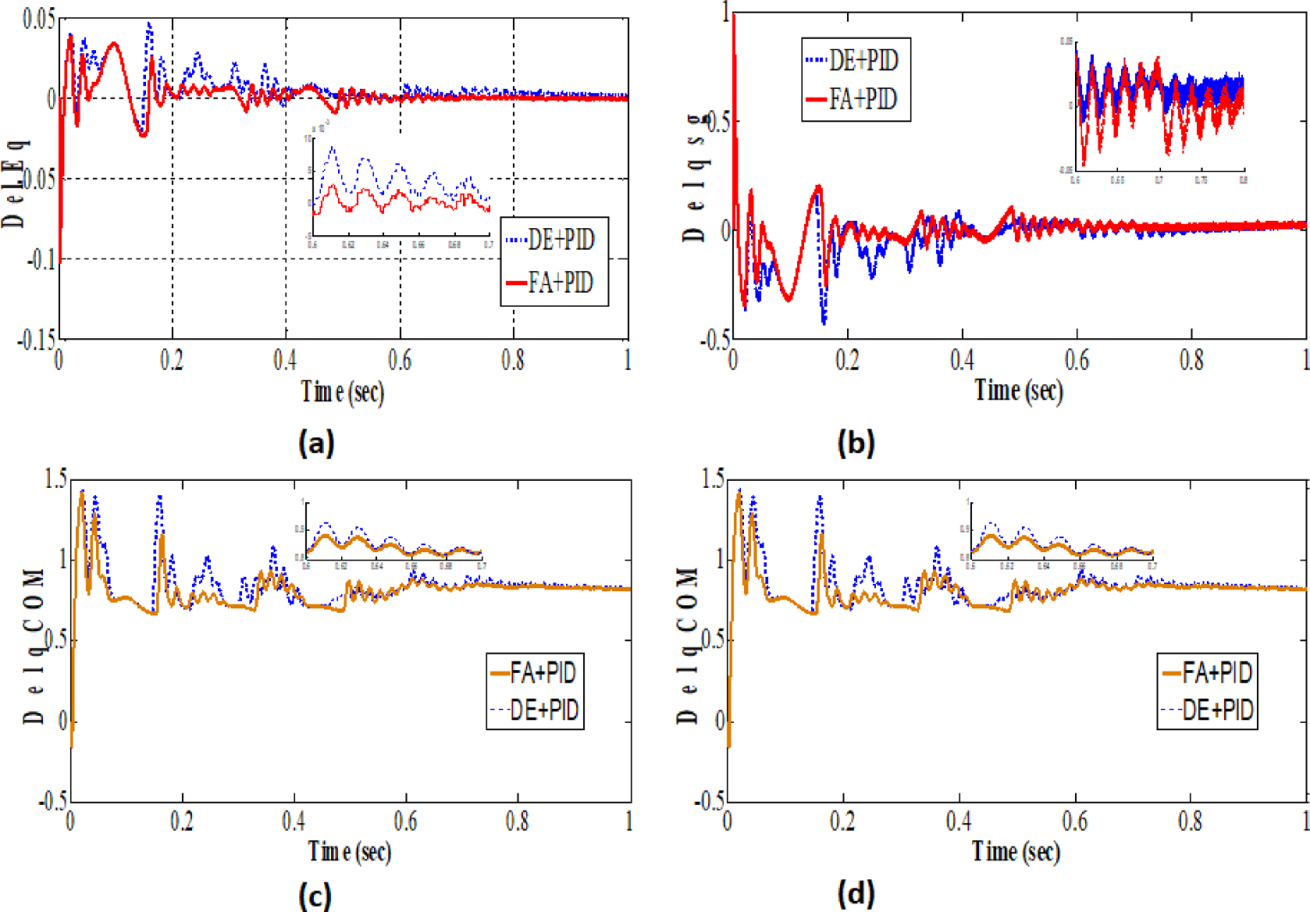
(**a-d**). Parameter outcome with DEA and FA based optimization.

A comparison study was performed to evaluate the dynamic responses of tidal turbines utilizing DFIG and DDPMSG systems, in connection with custom power devices such as UPFC. The investigation of optimized outcomes has been conducted using a UPFC controller based on DEA, FA & Hybrid FA. During the observation, it was noted that the transient responses of the DDPMSG system with UPFC demonstrate superior damping in contrast with the DFIG-based Tidal hybrid PS[Fig. 12]. The performance of DDPMSG, a high-performance system, has been enhanced by including algorithms based on DEA and FA. Further a hybrid FA based UPFC has been found to yield superior damping characteristics compared to FA and DEA based UPFC controller. One might conclude that the dynamic reactions of a Tidal-powered hybrid PS with UPFC surpass those of a DFIG-based system, as depicted in Fig. 13(a-c).

**Fig. 14:**
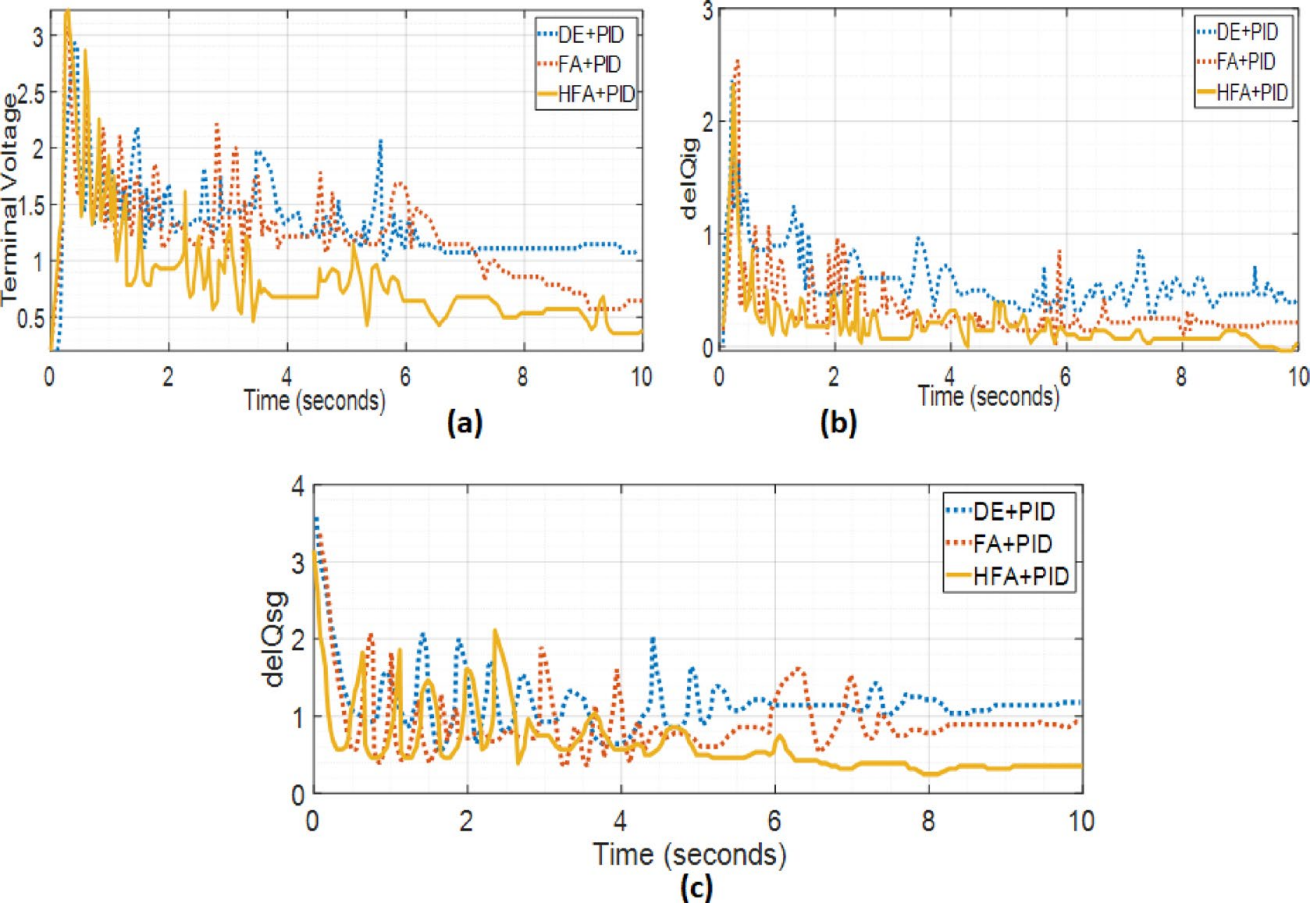
(**a-c**). Analyzing the response duration for various parameters within the hybrid system.

The transient responses depicting a 5% incremental load rise in the Tidal Turbine-driven hybrid PS with UPFC are illustrated in Fig. 14(a-f). It has been observed that in order to address the reactive power mismatch during transients, a larger gain is required to compensate for the reduced size of the UPFC and Tidal power generating unit. The change in peak value of terminal voltage is dependent on the dimensioning of the tidal turbine. The outcomes of the optimization using hybrid FA, DEA and FA have been compared with alternative methodologies, as depicted in Fig. 13 and summarized in Table 2.

**Fig. 15:**
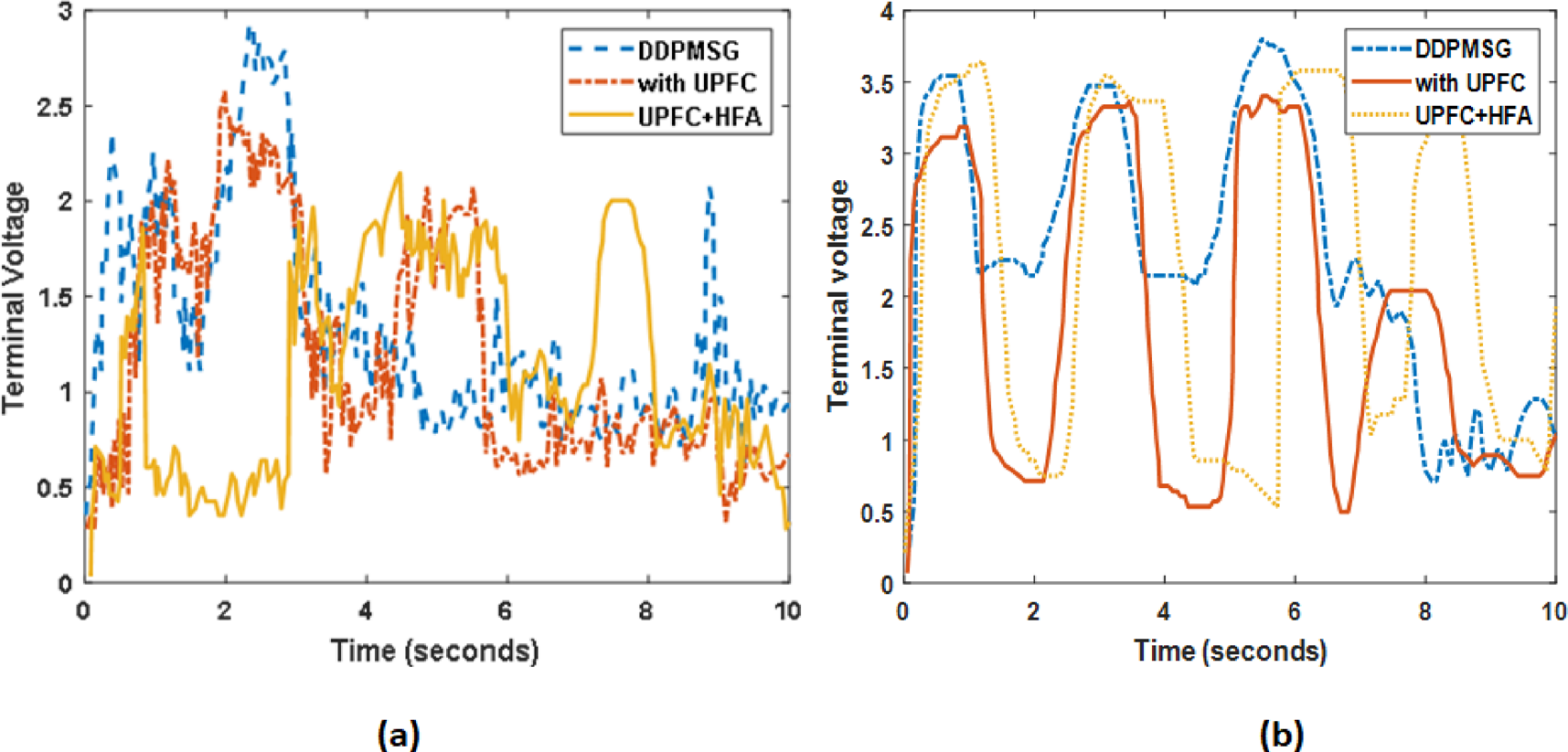
(**a**).uncertain wind (**b**).uncertain tidal.

The PI and PID gains of the UPFC in DDPMSG based system have been optimized through tuning, with a 5% increment increase in load together with tidal energy. The optimal parameters for harnessing tidal energy using Optimization techniques have been demonstrated in [Figure13(a-d)] and [Figure14 (a-c)]. The optimized values have been obtained through the utilization of DEA, FA and hybrid FA in the context of UPFC. The voltage deviation peak values and settling time during observation exhibit variation as the size of the DFIG/DDPMSG based turbine is reduced as illustrated in Figure 15 (a-b)..

### A. Stability study

**Fig. 16:**
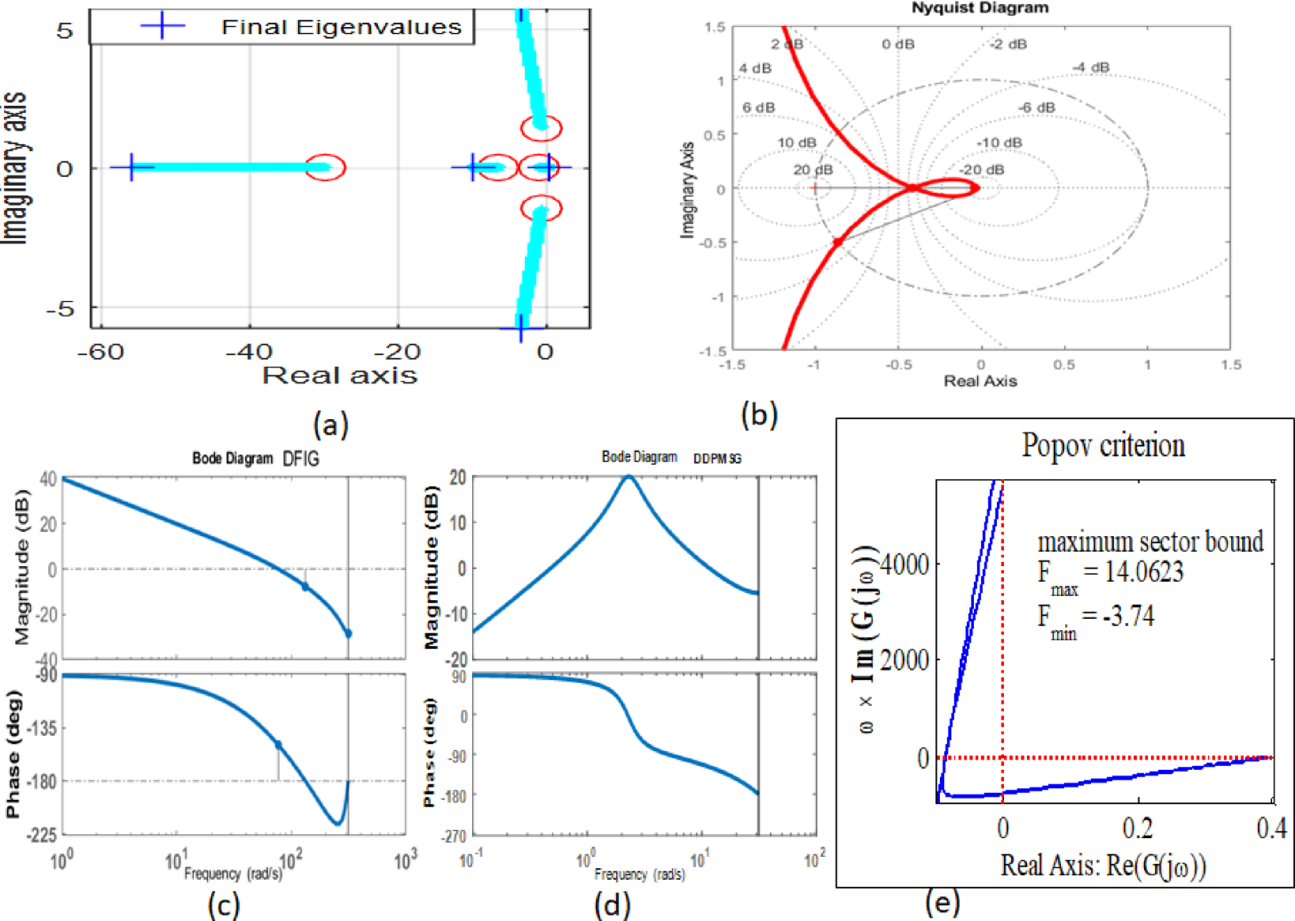
**(a)** Eigen value plot with DDPMSG, (**b**) Nyquist plot with DDPMSG, (**c**) Bode plot DFIG, (**d**) Bode plot DDPMSG, (**e**) Popov criteria.

Moreover, the examination of stability in the Tidal-powered hybrid PS with DFIG and DDPMSG may be scrutinized through diverse techniques, encompassing Bode, Nyquist, circle, and Popov methods, depicted in Fig. 16(a-e). The second mentioned method (Nyquist) states, this is going to attain a stable state if the response coefficient, which is K, positions the point $\frac{-1}{K}$remains into the positive side of the consistency boundary. The parameter’s identification scope of K is crucial for ensuring the Tidal system robustness. The suggested control approach has been formulated to satisfy the existing range of K. The value of K is limited by the fact that it is written like -inf < K<61.136. Furthermore, while taking into account other notable stability criteria such as Popov stability, it can be observed that the Popov line retains a random orientation. The ${F}_{min}$and ${F}_{max}$ values are determined by the intersection of the Popov line with the real axis. The investigation conducted by Circle and Popov provides valuable perspectives on pinpointing the scope of K and the criteria for sector delineations concerning voltage stability^33^.

When the UPFC-based controller is turned on, these dominant eigenvalues move deeper into the left-half plane. This means that oscillations are damped better and fade faster. The controller’s synchronized series-shunt action is what caused this change. It changes the flow of power and voltage in response to changes in renewable input.

The Nyquist analysis has also been expanded to show how the proposed controller boosts gain and phase margins. The regulated system is clearly apart from the critical point (− 1, j0), which shows that it is more robust to changes in parameters and outside disturbances. The higher Nyquist margins are due to the controller’s capacity to change reactive power and line impedance in real time, which keeps the power transfer characteristics stable even when the operating conditions change.

**Fig. 17:**
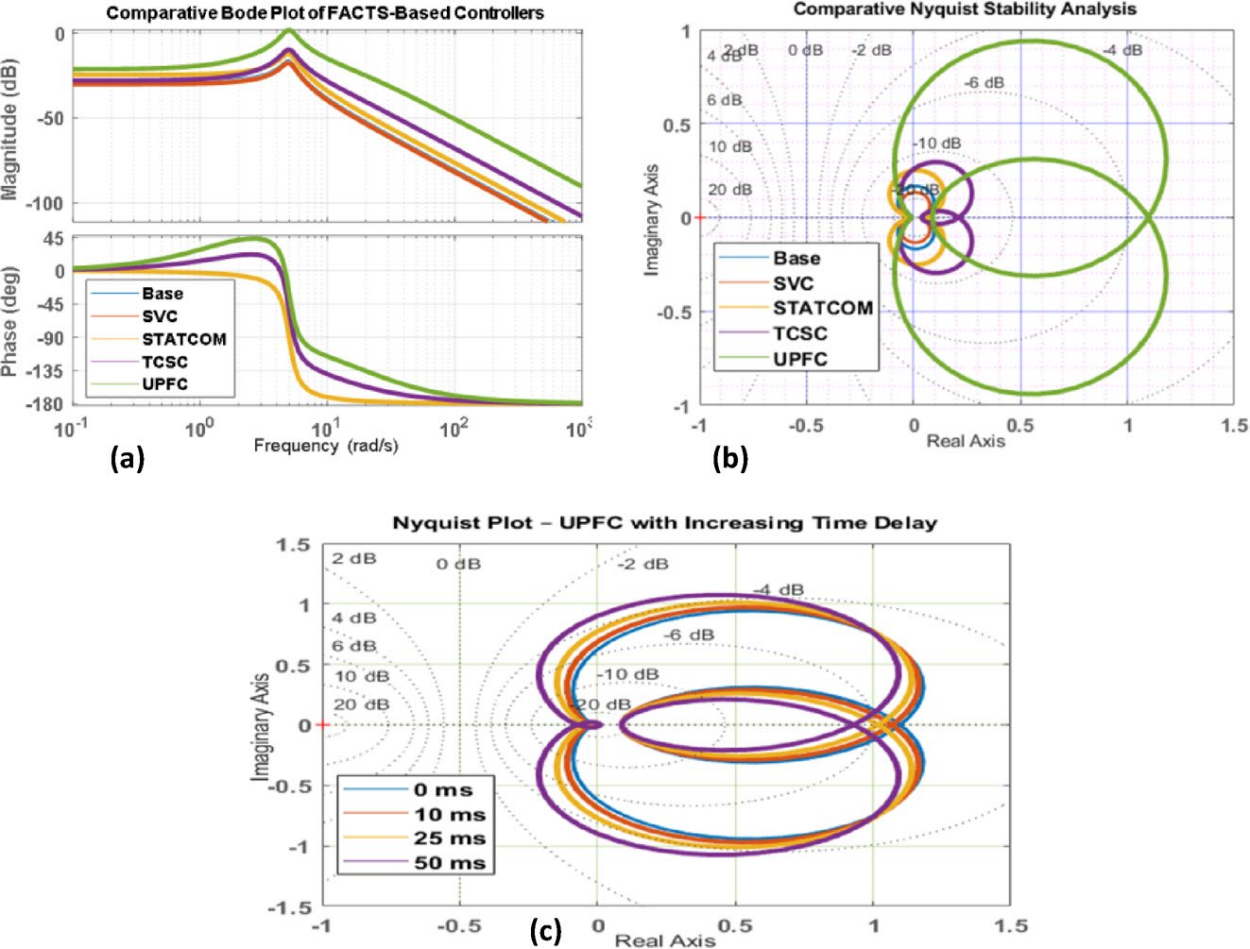
(**a-c**).comparison of Bode and Nyquist plots with different FACTS controllers.

Using Bode and Nyquist plots to undertake frequency-domain stability analysis shows that the UPFC-controlled system has far higher gain and phase margins than compensation based on SVC, STATCOM, or TCSC[Fig. 17(a-c)]. The Nyquist trajectories for the UPFC stay far away from the critical point, which shows that the system is stable even when the controller gain is high. Root locus analysis shows that UPFC adds more dampening torque, which moves the dominant poles deeper into the left-half plane. These results confirm that UPFC is better at dampening oscillations and is more stable for hybrid tidal-wind systems.

**Table 3:** Damping coefficient and characteristic values of the suggested hybrid PS.

Tidal system (DFIG)			Tidal system (DDPMSG)		
Eigen value	Damping	Frequency	Eigen value	Damping	Frequency
−3.82e-2	1.00e + 00	3.82e-02	−3.82e-02	1.00e + 00	3.82e-02
−4.84e + 00	1.00e + 00	4.84e + 00	−4.84e + 00	1.00e + 00	−4.84e + 00
−1.17e + 1 + 2.36e + i	3.27e-01	3.23e + 01	−1.06e + 1 + 3.05e + i	3.29e-01	3.27e + 01
−1.17e + 1–2.36e-i	3.27e-01	3.22e + 01	−1.06e + 1 + 3.04e + i	3.35e-01	3.24e + 01
−4.43e + 01	1.00e + 00	4.44e + 01	−1.17e + 1 + 3.06e + i	3.35e-01	3.24e + 01
−1.44e + 02	1.00e + 00	1.44e + 02	−1.17e + 1 + 3.05e + i	3.58e-01	3.27e + 01
−6.13e + 02	1.00e + 00	6.13e + 02	−4.47e + 1	1.00e + 00	4.47e + 01
−7.47e + 04	1.00e + 00	7.47e + 04	−1.46e + 2	1.00e + 00	1.46e + 02
			−6.17e + 2	1.00e + 00	6.18e + 02
			−7.89e + 4	1.00e + 00	6.18e + 02

Eigenvalues Table 3 provides insight into the stability of a hybrid PS that relies on tidal energy, particularly when subjected to load fluctuations and unknown tidal power inputs. Upon conducting observations, it has been determined that eigenvalues consistently reside towards the negative side of the imaginary line within the s-plane. The eigenvalues of the DDPMSG system are observed to be located to the left of the eigenvalues of the DFIG-based Tidal hybrid PS, suggesting an improvement in damping as well as stability characteristics[Table 4].

The participation matrix Table IV illustrates the connection between the chosen state variables and their respective eigenvalues. The relationship between the right and left eigenvectors is typically indicative of an association.

$$\left[P\right]=\left[P1\cdots\cdots \cdots \cdots Pn\right]$$

$${p}_{i}=\left[\begin{array}{c}{p1}_{i}\\{p2}_{i}\\*\\*\\{pk}_{i}\end{array}\right]\left[\begin{array}{c}\begin{array}{c}{f1}_{i\phi i1}\\{f2}_{i\phi i 2}\\*\end{array}\\*\\{fk}_{i\phi ik}\end{array}\right]$$

${f}_{ki}$ represent the k-th row and i-th column of the matrix [$\phi$].

**Table 4:** Analysis of involvement coefficients of DFIG &DDPMSG.

Participation factor Matrix (DFIG)
$$\left[ {\begin{array}{*{20}{c}} { - 0.01004}&{0.104312\,}&{ - 0.3212}&{ - 0.899106\,}&{ - 0.87810}&{\,\,\,0.52713}&{ - 0.00305}&{.3415} \\ { - 2.71e - 11}&{ - 5.014e - 06}&{ - 2.7e - 04}&{0.00123}&{0.01245}&{0.00043}&{ - 0.00076}&{0.1589} \\ {4.7815}&{0.000413}&{0.00143}&{0.001032}&{ - 0.0017}&{ - 0.0451}&{ - 0.04509}&{0.0421518} \\ {8.78e - 07}&{0.8942}&{0.6915}&{ - 7.07e - 04}&{ - 7.078 - 04 - 0.0069i}&{ - 0.2968}&{0.28412}&{ - 0.2903} \\ { - 1.169e - 04}&{ - 0.0210}&{0.4215}&{ - 0.00043}&{ - 0.00397 - 0.0069i}&{ - 0.2673}&{0.278013}&{ - 0.2892} \\ {0.09835}&{0.95546}&{0.2856}&{ - 0.00298}&{ - 002987 - 0.015i}&{ - 0.5986}&{0.78277}&{ - 0.5898} \\ { - 0.8998}&{ - 0.0678}&{ - 0.0458}&{ - 0.00049 - 0.00029i}&{ - 0.000495+0.0029i}&{0.0298}&{ - 0.00289}&{ - 0.00981} \end{array}} \right]\left[ \begin{gathered} \Delta I_{{dr}}^{{ref}} \hfill \\ \Delta {I_{dr}} \hfill \\ \Delta V \hfill \\ \Delta {E_{fd}} \hfill \\ \Delta {V_a} \hfill \\ \Delta {V_f} \hfill \\ \Delta E_{q}^{\prime } \hfill \\ \end{gathered} \right]$$
Participation factor Matrix (DDPMSG)
$$\left[ {\begin{array}{*{20}{c}} { - 0.0000i}&{ - 0.0000i}&{0.0001 - i}&{ - .4577+.162i}&{ - .4577+.162i}&{ - 0.0049+0.0i}&{ - 0.0004 - 0.0i}&{0.0253 - 0.0i}&0&0 \\ { - 0.0000i}&{0.0001+0.00i}&{0.503 - 0.139i}&{0.503+0.139i}&{0.5030.139i}&{0.0001+0.0i}&{0.0001+0.0i}&{0.0253 - 0.0i}&0&0 \\ { - 0.0000i}&{ - 0.0000i}&{ - 0.00 - 0.0i}&{ - .0122 - .018i}&{ - 0.0000 - 0.0i}&{0.0002+0.0i}&{ - 0.0033 - 0.0i}&{0.9748+0.0i}&0&0 \\ { - 0.0000i}&{ - 0.0001+0.0i}&{ - 0.0074+0.0i}&{ - 0.082+0.03i}&{0.0074+0.0i}&{0.0702+0.0i}&{ - 0.9452+0.0i}&{0.0016 - 0.0i}&0&0 \\ { - 0.0000i}&{0.9454+0.00i}&{.1639 - 0.0i}&{ - 0.004 - 0.01i}&{.1639 - 0.0i}&{ - 0.1109+0.0i}&{ - 0.0003 - 0.0i}&{ - 0.0000+0.0i}&0&0 \\ { - 0.0000i}&{0.032 - 0.0i}&{1.3622 - o.ooi}&{0.0012 - 0.6i}&{1.3622 - 0.00i}&{ - 0.3958+0.0i}&{ - 0.0009 - 0.0i}&{ - 0.0000+0.0i}&0&0 \\ { - 0.0000i}&{.0217+.0i}&{ - 0.519 - 0.0i}&{0.066 - 0.044i}&{0.5199 - 0.0i}&{1.4506+0.0i}&{0.0597 - 0.0i}&{ - 0.0039 - 0.0i}&0&0 \\ { - 0.0000i}&{1.0+0.00i}&{ - 0.02+0.0i}&{0.0000+0.0i}&{ - 0.00+0.0i}&{ - 0.00 - 0.0i}&{ - 0.00 - 0.0i}&{ - 0.00+0.0i}&{ - 0.00 - 0.0i}&0 \\ 0&0&0&0&0&0&0&0&0&{ - 0.5 - 0.1487i} \\ 0&0&0&0&0&0&0&0&0&{0.5+0.1487i} \end{array}} \right]\left[ \begin{gathered} {\omega _r} \hfill \\ {i_{ds}} \hfill \\ {i_{qs}} \hfill \\ {V_{dc}} \hfill \\ {X_1} \hfill \\ {X_2} \hfill \\ {X_3} \hfill \\ {X_4} \hfill \\ {X_5} \hfill \\ {X_6} \hfill \\ \end{gathered} \right]$$

**Fig. 18:**
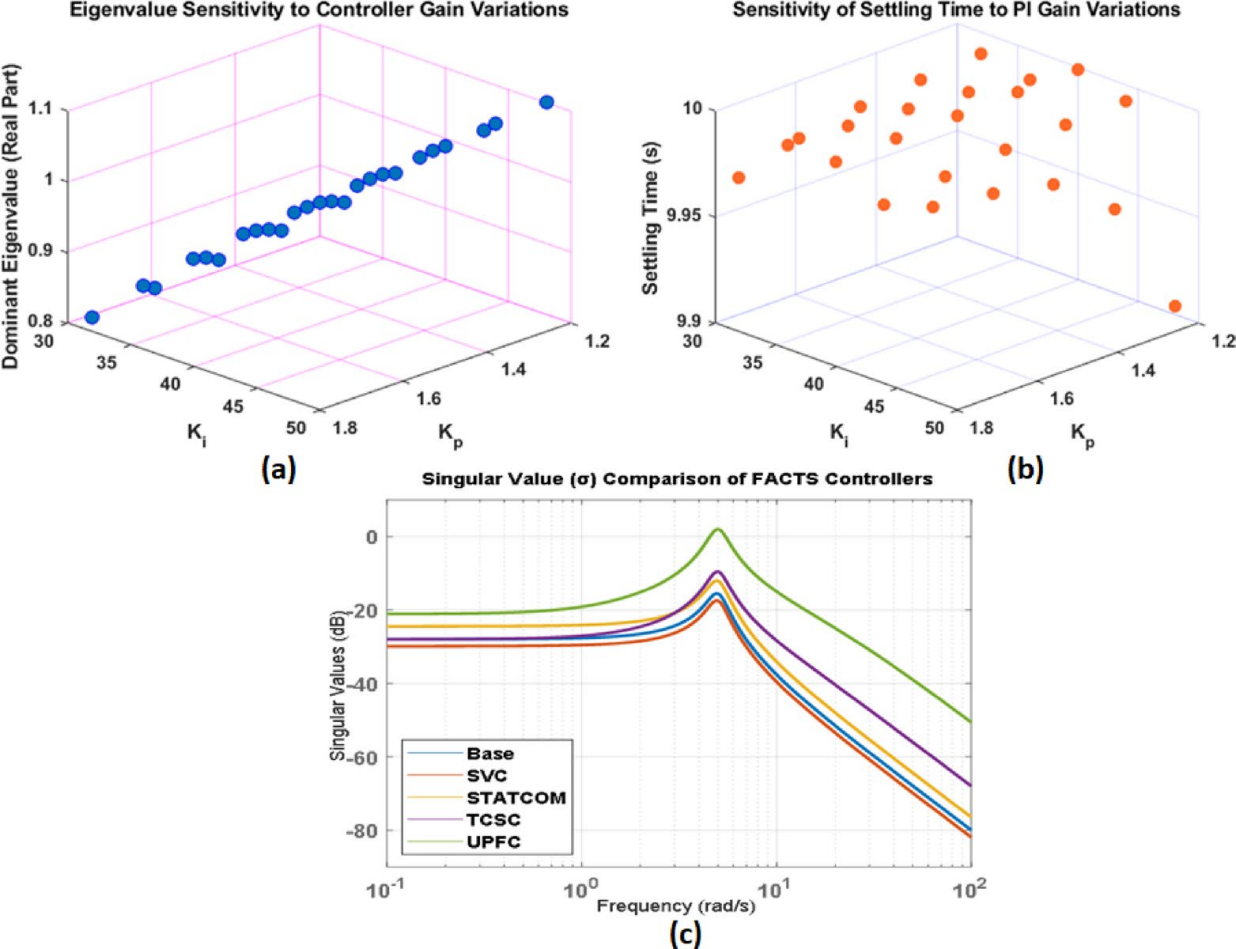
(**a-c**).sensitivity analysis of the proposed controller.

A sensitivity analysis was performed to evaluate the resilience of the proposed controller in the face of parameter ambiguity. We changed the proportional and integral gains by ± 20% of their nominal values on their own, and then settling time, rising time, and peak overshoot as performance indices are used to see how the closed-loop responses changed. The results[Fig. 18(a-c)] show that the system stays stable for all tested gain increases, with only small changes in how it responds to changes in the transient response. There were no sustained oscillations or instability, which shows that the controller is quite resilient to changes in gain and modeling errors.

### B. Experimental study through OPAL-RT

To evaluate the effectiveness of the suggested control strategy and the performance of the corresponding system model, a real-time analysis is conducted utilizing the OPAL-RT 5142 platform. Subsequent to this simulation, the results are reviewed and discussed in the following subsection. To gauge the efficiency of the proposed hybrid power system, a hardware in loop simulation (HILS) is executed employing the Opal-RT OP5142 simulator (as shown in Fig. 17(a-b)), alongside a digital signal processor controller board featuring a TIF28335 microcontroller unit. The comprehensive diagram of the HILS illustrates the Tidal power plant, diesel plant, and UPFC plant simulated in real-time with a sampling interval of 20µs. Control algorithm and optimization techniques are developed within the Microcontroller unit accordingly. Inputs including measurements and PWM signals are received by the controller board, sourced from the Opal-RT simulator via its input/output interfaces.

**Fig. 19:**
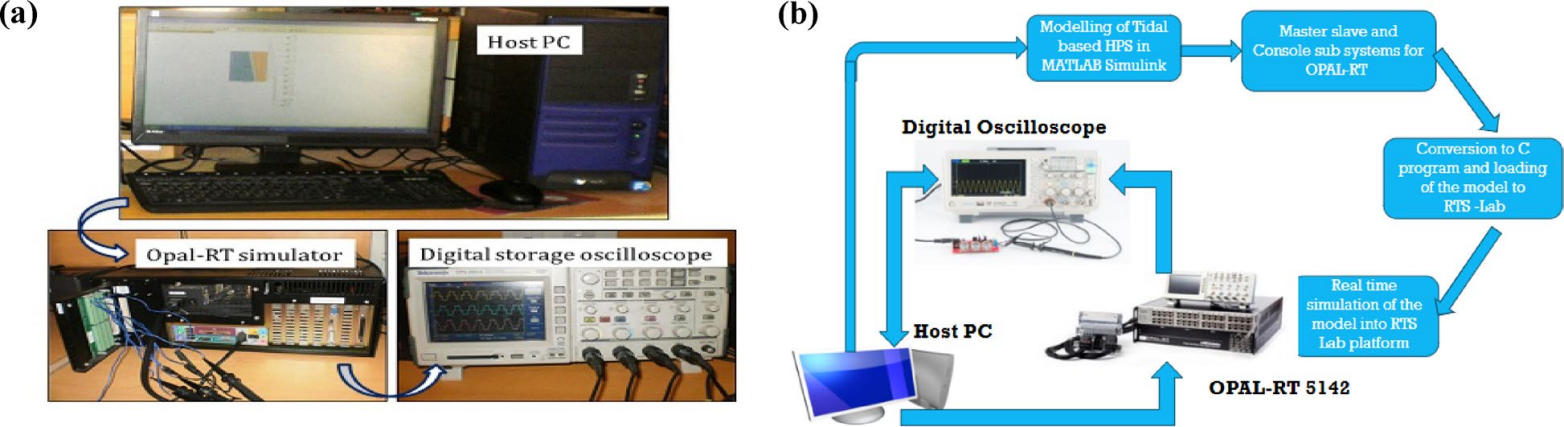
(**a**). Experimental study through OP 5142 for stability analysis, (**b**)Structure of the hardware in the loop simulation.

The real-time simulation runs with a fixed time step of 50 µs, which is enough to accurately depict both quick converter dynamics and slower electromechanical oscillations. At 10 kHz, the controller sampling is in sync with the OPAL-RT solver, which makes sure that the execution is deterministic and the numbers are stable. OPAL-RT’s low-latency I/O interface is used to connect the real-time model and the controller. The measured end-to-end communication latency stays below 1 ms, which is well within the constraints for wide-area and converter-based control applications. To make sensor behavior more realistic, OPAL-RT signal conditioning blocks include measurement noise and quantization effects.

**Fig. 20:**
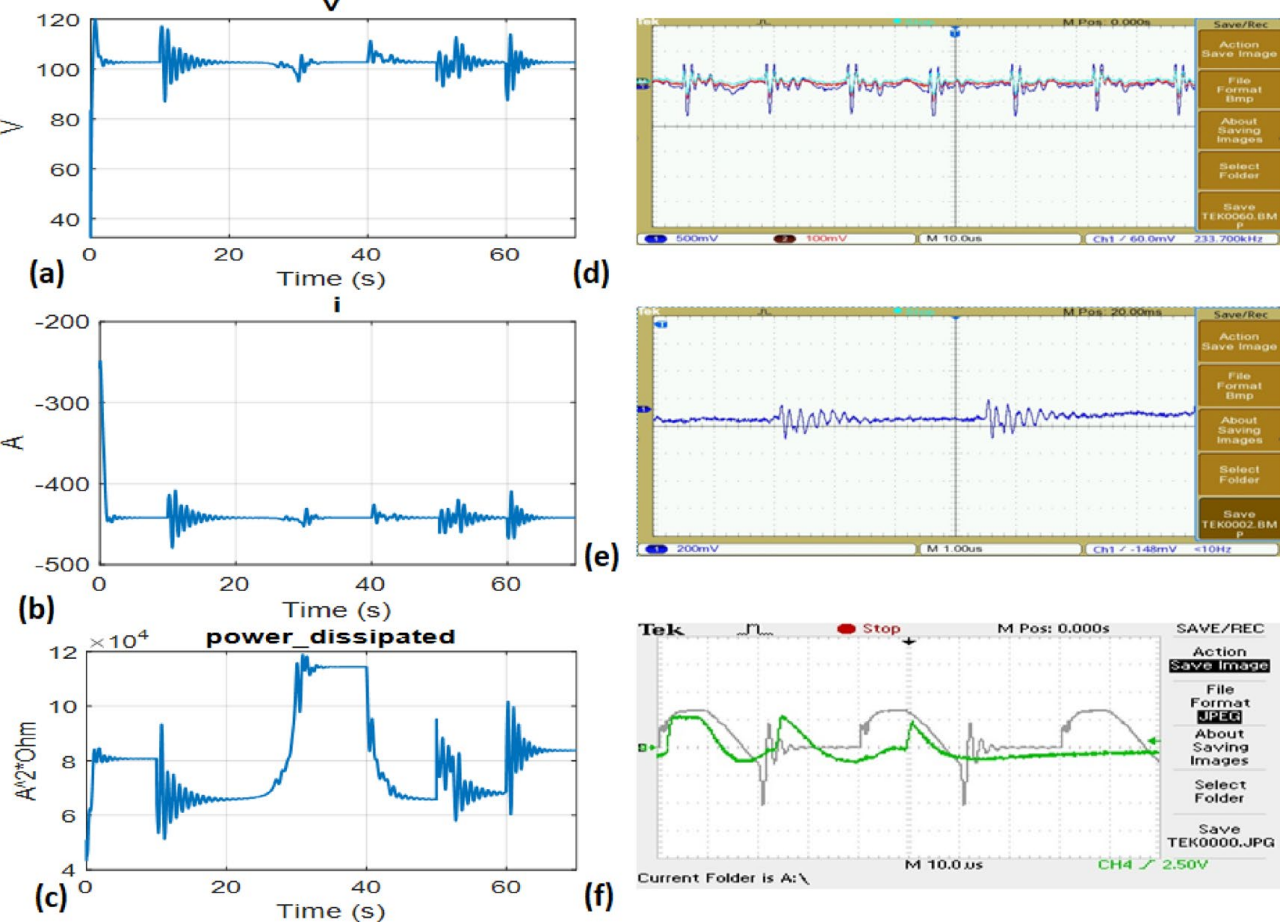
(**a-c**). Simulation results of Terminal Voltage, Terminal current and power output, (**d-f**). Real time results of Terminal voltage, current and power output.

The tests conducted in this part utilizing the OPAL RT platform Fig. 19(a-b), employing various optimization strategies, demonstrate a high level of robustness in performance when subjected to parameter variations. The dynamic performance is then displayed in Fig. 20(a-f) after the model has been run with a 5% load change. The application of a hybrid FA-optimized PID controller has demonstrated improved system dynamics in comparison to the other two controllers, particularly regarding stabilization duration, excessive deviation, and insufficient response.The experimental findings demonstrated that the utilization of the FA yielded a notable rate of convergence and a robust capacity for exploration. Conversely, the DEA exhibited proficiency in exploitation through the effective use of mutation and crossover operators.

## Conclusion

This research examines the stability and optimization of the hybrid PS using DEA and FA based UPFC. It is suggested to develop a novel hybrid FA that combines certain advantageous aspects of both the FA and DEA. Based on the theoretical analysis and the problem-solving capabilities of metaheuristic algorithms, it can be concluded that the hybrid FA offers three distinct advantages or enhancements. Firstly, hybrid FA demonstrates an improved equilibrium between exploration and exploitation by employing a parallel utilization of FA and DEA, along with the sharing of population information. Further the UPFC has emerged as a leading bespoke power device, effectively enhancing the Tidal system resilience as well as demonstrating its robustness in administering reactive power. The analysis of fine-tuning and the result of the system at both steady and temporary states are conducted using the DEA as well as FA. The findings suggest that the FA-based DDPMSG exhibits greater stability, as seen by improved performance in stabilization and ascent durations, maximum overshoot, as well as attenuation. Additionally, this research integrates a live experimental investigation to authenticate the simulation results using the OPAL-RT Platform. The effectiveness of the proposed controllers is evaluated through stability analysis employing Eigen and Nyquist plots. The proposed control methodology in the connected power system is confirmed using an OPAL-RT 5142.

## Appendix-I

**Table:** 

Parameter	Symbol	Typical Value	reference
Rated turbine diameter	R	10	m
Seawater density	ρ	1025	kg/m³
Optimal tip-speed ratio	λₒₚₜ	6–8	–
Rated speed	ωᵣ	120	rad/s
PMSG pole pairs	p	40	–
Stator resistance	Rs	0.5	Ω
Inductance	Ld = Lq	15	mH
Magnet flux linkage	ψf	0.6	Wb
DC-link capacitance	Cdc	2200	µF
Converter filter inductance	Lf	5	mH
Converter filter resistance	Rf	0.05	Ω
Inertia constant	J	15	kg·m²
Damping coefficient	B	0.02	N·m·s

**Appendix-II.** Unimodal benchmark function.

**Table:** 

Function name	Function	D	S	${F}_{min}$
Sphere	${f}_{1}\left(x\right)=\sum_{i=1}^{D}{X}_{i}^{2}$	30	${\left[-\mathrm{100,100}\right]}^{D}$	0
Schwefel’s 2.22	${f}_{2}\left(x\right)=\sum_{i=1}^{D}{X}_{i}+\prod_{i=1}^{D}\left|{X}_{i}\right|$	30	${\left[-\mathrm{10,10}\right]}^{D}$	0
Schwefel’s 1.20	${f}_{3}\left(x\right)=\sum_{i=1}^{D}{\left(\sum_{j=1}^{D}{X}_{j}\right)}^{2}$	30	${\left[-\mathrm{100,100}\right]}^{D}$	0
Schwefel’s 2.21	${f}_{4}\left(x\right)={max}_{i}\left\{\left|{X}_{i}\right|,1\le i\le D\right\}$	30	${\left[-\mathrm{100,100}\right]}^{D}$	0
Rosenbrock	${f}_{5}\left(x\right)=\sum_{i=1}^{D-1}100{\left({X}_{i+1}-{X}_{i}^{2}\right)}^{2}+{\left({X}_{i}-1\right)}^{2}$	30	${\left[-\mathrm{30,30}\right]}^{D}$	0
Step	${f}_{6}\left(x\right)=\sum_{i=1}^{D}{\left({X}_{i}+0.5\right)}^{2}$	30	${\left[-\mathrm{100,100}\right]}^{D}$	0
Quartic Noise	${f}_{7}\left(x\right)=\sum_{i=1}^{n}i{X}_{i}^{4}+random\left[0,\left.1\right)\right.$	30	${\left[-\mathrm{1.28,1.28}\right]}^{D}$	0

**Appendix-III**. Multimodal benchmark function.

**Table:** 

Function name	Function	D	S	${F}_{min}$
Schwefel’s 2.26	${f}_{8}\left(x\right)=\sum_{i=1}^{D}-{X}_{i}sin\left(\sqrt{\left|{X}_{i}\right|}\right)$	30	${\left[-\mathrm{500,500}\right]}^{D}$	−12569.5
Restrigin	${f}_{9}\left(x\right)=\sum_{i=1}^{D}\left[{X}_{i}^{2}-10cos\left(2\pi{X}_{i}+10\right)\right]$	30	${\left[-\mathrm{5.12,5.12}\right]}^{D}$	0
Ackley	${f}_{10}\left(x\right)=-20exp\left(-0.2\sqrt{\frac{1}{n}\sum_{i=1}^{n}{X}_{i}^{2}}\right)-exp\left(\frac{1}{n}cos\left(2\pi\left({X}_{i}\right)\right)\right)+20+e$	30	${\left[-\mathrm{32,32}\right]}^{D}$	0
Griewank	${f}_{11}\left(x\right)=\frac{1}{4000}\sum_{i=1}^{n}{X}_{i}^{2}-\prod_{i=1}^{n}cos\left(\frac{{X}_{i}}{\sqrt{i}}\right)+1$	30	${\left[-\mathrm{600,600}\right]}^{D}$	0
Pendlized	$$\begin{aligned}{f}_{12}\left(x\right)&=\sum_{i=1}^{D}u\left({X}_{i},\mathrm{10,100,4}\right)\\&+\frac{\pi}{D}\left\{10{sin}^{2}\left(3\pi{Y}_{1}\right)\sum_{i=1}^{D-1}{\left({Y}_{i}-1\right)}^{2}\right.\\& \left.\left[1+{sin}^{2}\left(3\pi{Y}_{i+1}\right)+{\left({Y}_{D}-1\right)}^{2}\right]\right\}\\&\quad{Y}_{i}=1+\frac{1}{4}\left({X}_{i}+1\right)u\left({X}_{i},a,k,m\right)\\&=\left\{\begin{array}{cc}k{\left({X}_{i}-1\right)}^{m}&{X}_{i}>a\\0&-a\le{X}_{i}\le a\\k{\left(-{X}_{i}-1\right)}^{m}&{X}_{i}<-a\end{array}\right.\end{aligned}$$	30	${\left[-\mathrm{50,50}\right]}^{D}$	0
Generalized Pendlized	$$\begin{aligned}{f}_{13}\left(x\right)&=\sum_{i=1}^{D}u\left({X}_{i},\mathrm{5,10,4}\right)\\&\quad+\frac{1}{10}\left\{{sin}^{2}\left(3\pi{X}_{1}\right)\sum_{i=1}^{D-1}{\left({X}_{i}-1\right)}^{2}\left[1+{sin}^{2}\left(3\pi{X}_{i+1}\right)\right.\right.\\&\qquad\left.\left.{\left({X}_{D}-1\right)}^{2}\left[1+{sin}^{2}\left(2\pi{X}_{D}\right)\right]\right]\right\}\\&\quad u\left({X}_{i},a,k,m\right)=\left\{\begin{array}{cc}k{\left({X}_{i}-1\right)}^{m}&{X}_{i}>a\\0&-a\le{X}_{i}\le a\\k{\left(-{X}_{i}-1\right)}^{m}&{X}_{i}<-a\end{array}\right.\end{aligned}$$	30	${\left[-\mathrm{50,50}\right]}^{D}$	0

**Appendix-IV**. Results of Multimodal benchmark functions.

**Table:** 

Function	Statistics	Hybrid FA	Mean Value	FA	Mean Value	DEA	Mean Value
${f}_{8}$	Minimum	−10,596	−9469.5	−5627.9	−5016.3	−10,001	−9020.8
	Maximum	−8424.1		−4515.8		−7093.8	
	Mean	−9469.5		−5016.3		−9020.8	
	Standard	−515.47		260.0659		515.74	
${f}_{9}$	Minimum	3.9798	9.3858	143.8889	175.9112	17.909	30.15
	Maximum	15.919		196.3629		44.773	
	Mean	9.3858		175.9112		30.15	
	Standard	3.0436		12.24334		7.1079	
${f}_{10}$	Minimum	7.99E-15	1.25E-14	4.3632e-05	3.1272e-04	4.75E-07	7.10E-06
	Maximum	1.51E-14		0.0031		6.15E-05	
	Mean	1.25E-14		3.1272e-04		7.10E-06	
	Standard	3.36E-15		5.4874e-04		1.47E-05	
${f}_{11}$	Minimum	0	0	1.8299e-07	0.0132	3.74E-12	0.013444
	Maximum	0		0.1005		0.046483	
	Mean	0		0.0132		0.013444	
	Standard	0		0.0220		0.012311	
${f}_{12}$	Minimum	1.57E-32	1.57E-32	1.8989e-09	2.2768e-04	2.34E-12	0.024188
	Maximum	1.57E-32		0.0036		0.31096	
	Mean	1.57E-32		2.2768e-04		0.024188	
	Standard	5.57E-48		6.8779e-04		0.064897	
${f}_{13}$	Minimum	1.35E-32	1.35E-32	6.6202e-08	1.1956e-05	2.42E-11	0.0032963
	Maximum	1.35E-32		7.1684e-05		0.010987	
	Mean	1.35E-32		1.1956e-05		0.0032963	
	Standard	5.57E-48		1.7650e-05		0.0051211	

## Data Availability

The data will be made available from the corresponding author upon reasonable request.

## Abbreviations

${P}_{IG}$: Real power DFIG/DDPMSG
${Q}_{DFIG}$: Reactive power DFIG
${P}_{SG}$: Real power SG
${Q}_{SG}$: Reactive power SG
${E}_{M}$: Energy storage (DFIG)
${\varDelta E}_{M}$: Variation in change storage
Q_UPFC_: Reactive power UPFC
${\varDelta Q}_{UPFC}$: Variation in Reactive Power
${K}_{\propto},{K}_{V}$: Exciter gain, Gain of energy balance
${Q}_{DDPMSG}$: Reactive power/DDPMSG
${T}_{\propto},{T}_{r},{T}_{s}$: Exciter time, Time-to-rise, Stabilization time constants
${X}_{d}$: Direct axis reactance of SG
${T}_{e}{,T}_{m}$: Electromagnetic variation, mechanical torque
$\varDelta$α: Phase angle modification
${\varDelta E}_{q}$: Change in internal armature voltage
${\varDelta E}_{fd}$: Change in voltage of Exciter
